# Phytohormones Regulate Accumulation of Osmolytes Under Abiotic Stress

**DOI:** 10.3390/biom9070285

**Published:** 2019-07-17

**Authors:** Anket Sharma, Babar Shahzad, Vinod Kumar, Sukhmeen Kaur Kohli, Gagan Preet Singh Sidhu, Aditi Shreeya Bali, Neha Handa, Dhriti Kapoor, Renu Bhardwaj, Bingsong Zheng

**Affiliations:** 1 State Key Laboratory of Subtropical Silviculture, Zhejiang A&F University, Hangzhou 311300, China; 2 School of Land and Food, University of Tasmania, Hobart, Tasmania 7005, Australia; 3 Department of Botany, DAV University, Sarmastpur, Jalandhar 144012, Punjab, India; 4 Plant Stress Physiology Laboratory, Department of Botanical & Environmental Sciences, Guru Nanak Dev University, Amritsar 143005, India; 5 Department of Environment Education, Government College of Commerce and Business Administration, Chandigarh 160047, India; 6 Mehr Chand Mahajan D.A.V. College for Women, Chandigarh 160036, India; 7 School of Bioengineering & Biosciences, Lovely Professional University, Phagwara 144411, India

**Keywords:** abscisic acid, brassinosteroids, cytokinins, ethylene, jasmonates, oxidative stress, plant hormones, salicylic acid

## Abstract

Plants face a variety of abiotic stresses, which generate reactive oxygen species (ROS), and ultimately obstruct normal growth and development of plants. To prevent cellular damage caused by oxidative stress, plants accumulate certain compatible solutes known as osmolytes to safeguard the cellular machinery. The most common osmolytes that play crucial role in osmoregulation are proline, glycine-betaine, polyamines, and sugars. These compounds stabilize the osmotic differences between surroundings of cell and the cytosol. Besides, they also protect the plant cells from oxidative stress by inhibiting the production of harmful ROS like hydroxyl ions, superoxide ions, hydrogen peroxide, and other free radicals. The accumulation of osmolytes is further modulated by phytohormones like abscisic acid, brassinosteroids, cytokinins, ethylene, jasmonates, and salicylic acid. It is thus important to understand the mechanisms regulating the phytohormone-mediated accumulation of osmolytes in plants during abiotic stresses. In this review, we have discussed the underlying mechanisms of phytohormone-regulated osmolyte accumulation along with their various functions in plants under stress conditions.

## 1. Introduction

Plants face a wide range of environmental stresses such as drought, salinity, heat, heavy metal, light, pesticide, and cold that hampers their physiological and cellular functioning [1,2,3,4]. Abiotic stresses are considered as the major threat to global agriculture systems [5]. Moreover, anthropogenic activities have also led to degradation of agricultural ecosystem and crop yield due to increased salt, drought, ozone, and metal stress [6,7,8,9,10,11]. These abiotic stresses cause huge crop loss by reducing yield by almost 50% in different crop plants [12] attributed to the increased generation of oxygen free radicals that induce oxidative stress in plants [13]. Abiotic stresses reduce plant development by affecting different biochemical and physiological mechanisms like photosynthesis, antioxidant systems, and hormonal signaling [14,15]. These environmental stresses stimulate complex responses in plants to prevent injury and increase their survivability under adverse conditions. Plants alter various cellular and molecular processes in reaction to abiotic stresses that eventually mount their growth and development [16,17]. Plants accumulate osmolytes or compatible solutes to protect the cellular machinery from various environmental stresses [18]. The most well-known osmolytes are glycine betaine (GB), sugars (mannitol, sorbitol, and trehalose), polyamines, and proline. These osmolytes get accumulated under various abiotic stresses and confer tolerance to cell without interfering with the cellular machinery of the plant [19,20,21]. Accumulation of sugars such as trehalose, mannitol, and galactinol under abiotic stress has been reported widely in plants, and several genes play important roles in biosynthesis of these organic solutes helping in the development of abiotic stress tolerance in transgenic plants [22]. Similarly, proline accumulation is also one of the mechanisms or responses in many plants under various stresses [6,20,21]. It is thus important to understand the mechanisms that control different processes and mechanism underlying abiotic stress tolerance in plants.

Phytohormones play an important role in various biochemical and physiological mechanisms in plants. Their role in alleviating abiotic stress is critical in providing tolerance to plants under adverse condition [5,9,10,11,23,24,25,26,27,28]. Since both osmolytes and plant hormones have been known to play major roles during challenging environments, it is therefore imperative to correlate both and to further elucidate the role of phytohormones in the regulation of osmolytes under abiotic stress.

## 2. Involvement of Osmolytes to Bestow Abiotic Stress Tolerance

**Figure 1:**
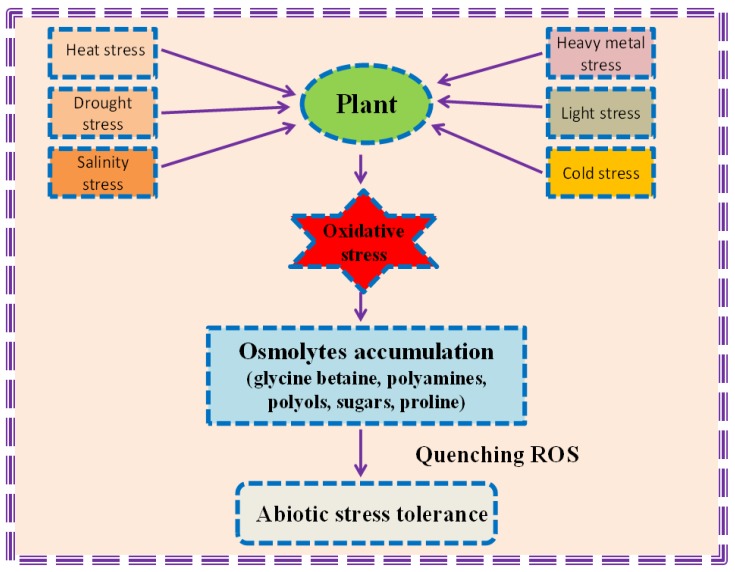
Schematic representation of plants response to various abiotic stresses and the role of osmolytes to counteract reactive oxygen species under stressful conditions.

Osmolytes are compatible osmoprotectant solutes that enhance the cell potential to maintain water without hampering the normal metabolism. The main purpose of these organic metabolites is to regulate osmotic adjustment. These osmotic solutes or osmoprotectants help the organisms to sustain severe osmotic stress during their life cycle [29]. These compounds stabilize the osmotic differences connecting cell’s surroundings and cytosol [30]. Besides, they protect plants from oxidative damage by inhibiting the production of ROS [31] (Figure 1). Osmolytes are neutral molecules that safeguard the proteins and other cell membranes against various stress factors on cellular metabolism [32]. These metabolites include proline, sucrose, polyols, trehalose, GB, and alanine betaine (Figure 1). These osmoprotectants conserve the cellular functions of the plant and is the main strategy adopted by plants to counterattack stresses. Many reports have suggested the role of osmolytes in drought [20,33,34], salinity [35], osmotic [36], heavy metal [37], temperature [38], light, and pesticide stress [39].

### 2.1. Drought Stress

The reduction in water content/potential of leaves is the most common indicators of drought stress in plants. It interrupts the physiological processes of plants like photosynthesis and causes death of the plants [40]. Din et al. [41] observed a sharp decline in the levels of chlorophyll in plants upon exposure to drought stress, which attributed to the effect on enzymes related to chlorophyll biosynthesis [42]. Plants have developed various mechanisms to counteract drought stress, one of them being accumulation of osmolytes or osmoprotectants in the plants in response to stress [9,20,43]. A range of osmotically active molecules such as sugars, proline, GB, and organic acids get accumulated to balance the water relations under water/drought stress. Due to aggregation of solutes in the cell under water stress, the osmotic potential of the cell becomes highly negative, which causes endosmosis of water into the cell and maintains the turgor of the cell. The above-mentioned osmotic adjustment is possible due to the accumulation of compatible solutes/osmolytes. Among them, proline is the most important osmolytes under drought stress. Alexieva et al. [44] observed increased levels of proline in pea cultivars under drought stress. Moreover, Yamada et al. [45] reported accumulation of free proline in a drought-tolerant variety of *Petunia hybrida* under drought stress.

Many reports have suggested the role of GB in providing tolerance towards drought stress in plants [46]. Higher accumulation of GB has been observed in cotton plants adapted to drought stress [47]. Citrulline, an amino acid isolated from watermelon, has been reported to impart drought tolerance in watermelon (*Citrullus lanatus*) [48]. Spermidine, a polyamine, enhanced plant resistance when applied exogenously to blueberry plants under drought stress [49]. Plants can tolerate drought stress mainly by osmotic adjustment and balancing the system of antioxidant defense that helps in scavenging ROS and providing stability to the cell membranes.

### 2.2. Salt Stress

Salt stress affects more than one third of the land mass on Earth [50]. It is an environmental limitation that has two main components: An osmotic component and an ionic component, which are related to reduced external osmotic potential of soil and increased accumulation of ions that poses threat at higher concentration. Salt stress causes lipid peroxidation, interrupts water balance, nutrient imbalance, disrupts the activity of various enzymes, and generates ROS species that eventually damage the photosynthetic apparatus [51,52,53,54]. Zhu [55] also reported reduced growth of plants upon exposure to salt stress due to instigating ion toxicity and oxidative stress. Plants tolerate salinity by accumulating low molecular weight osmolytes such as proline, GB, and polyamines that helps in maintaining stability of the membrane [56]. These osmoprotectants enhances the rate of germination, growth, and development thereby inducing tolerance in response to salt stress.

Many studies suggested that salt stress modulates the enzymes responsible in biosynthesis of proline and GB [57] and their increased concentration is correlated with increased stress tolerance [58]. Accumulation of proline is an adaptive mechanism against salt stress [59]. Khedr et al. [60] reported that proline regulates transcript levels of salt stress responsive proteins, resulting in improvement of plant adaption towards salt stress. Furthermore, activities of catalase and peroxidase enzymes were found to be higher in *Pancratium maritimum* in the presence of proline under salt stress [60]. It also increases the activity of enzymes that play roles in antioxidant defense systems as documented in *Nicotiana tobacum* [61]. GB is an organic and water-soluble molecule that protects the plants against salt stress [62]. It protects the plant by stabilizing proteins like RuBisCo [63] and reducing ROS production [64]. Lutts [65] observed that accumulation of GB improved the survivability and growth of salt-treated plants.

### 2.3. Temperature Stress

Plants are subjected to a range of temperature variations during changing seasons. High temperature stress results in a decreased amount of chlorophyll synthesis in plants [66], which is an indicator of stress in plastids [67]. For example, high temperature stress reduces activity of 5-aminolevulinate dehydratase (ALAD), an enzyme that helps in biosynthesis of chlorophyll [68]. Moreover, chilling stress also affects crop productivity by harming the metabolic machinery of plants. Chilling stress induces increased levels of ROS, especially H_2_O_2_ in plants, which might be responsible for adversely affecting growth and development of plants [69]. Plants have developed various strategies to tolerate temperature variations. One of the mechanisms adopted by plants under temperature stress is the accumulation of compatible solutes or osmolytes that have a protective role in plants. Kishor et al. [70] reported increased tolerance in plants exposed to cold stress due to elevated proline content. Exogenous application of proline at low concentration increased tolerance towards cold stress [71,72]. Many studies have reported the role of GB in protecting plants under temperature stress [22,73]. Due to advancement in technology, genes involved in synthesis of GB are now transferred in some non-accumulator plants that help plants survive under stresses. For example, *codA* gene provides tolerance towards cold stress in *Oryza sativa*, which protects the plant from membrane damage, enzyme, and ROS activity [38,74]. The ROS produced during the abiotic stress conditions damages PSII in chloroplast, however GB maintained the activity of proteins and protects the plant from stressful conditions.

### 2.4. Heavy Metal Stress

Heavy metal contamination has emerged as an extensive hazard in addition to drought, temperature, and salinity stress [6,75]. Due to increases in urbanization and industrialization, the use of heavy metals has increased at an alarming rate. The excessive generation of ROS in response to heavy metal stress induces oxidative stress in plants [8,76,77,78] harming the cellular [79], biochemical, and physiological mechanisms in plants [80,81]. Pb exposure caused reduction in leaf pigment concentration in *Coronopus didymus*, which might be due to decreased chlorophyll synthesis or its degradation in response to heavy metal stress [82,83]. Therefore, plants are engaged in various mechanisms to detoxify heavy metals and tolerate heavy metal stress. Dhir et al. [84] assessed osmolyte accumulation in *Salvinia natans* upon exposure of heavy metals. They noticed increased amounts of GB, sucrose, and mannitol in response to Cd, Cu, Ni, Zn, Pb, Fe, Mn, and Cr [84]. Likewise, Bhatti et al. [85] observed that exogenous application of GB increased the protection of wheat plants under heavy metal stress. They found that application of GB enhanced the root and shoot length, biomass and photosynthetic pigments, and promoted ROS scavenging as well as osmotic adjustments [85].

### 2.5. Light Stress

Various environmental factors regulate the growth and development of plants. The intensity of sunlight is an important factor that controls various metabolic activities in plants [86]. The fluctuations in solar radiations alter the light reactions of photosynthesis in thylakoid membranes [87]. Many reports pointed that light intensity affects stomatal density of mature leaves [88]. High light intensity specifically of the UV region damages the photosynthesis process due to overproduction of ROS, which damages the photosynthetic electron transport [89]. Wang et al. [90] reported increased accumulation of GB in response to high light stress in wheat, which aided in recovering chlorophyll content and the net photosynthetic rate in the plants by repairing PS II.

## 3. Role of Brassinosteroids in Regulation of Osmolytes Under Abiotic Stress

**Figure 2:**
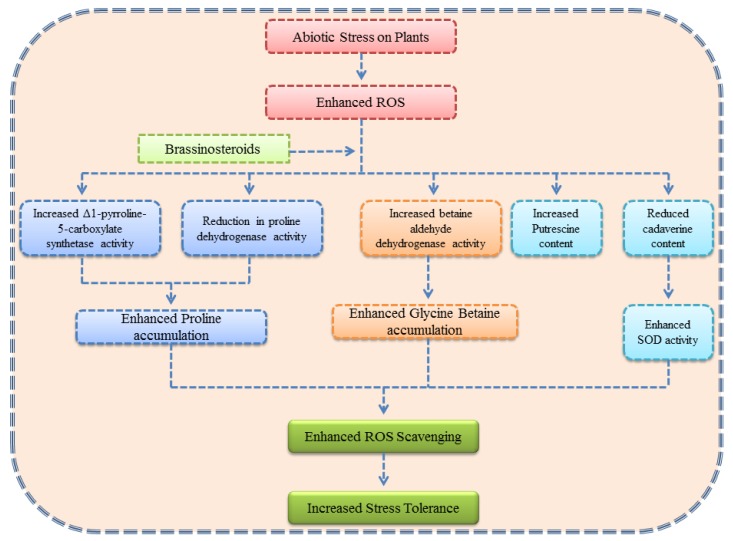
Schematic outline summarizing various effects of brassinosteroids on different osmolytes in plants under stressful conditions.

Brassinosteroids (BRs) are classified as the sixth class of plant hormones apart from the five classical hormones. These are of steroidal origin, which were first isolated from *Brassica* pollens [91]. Many studies on BRs later confirmed their ubiquitous presence in the plant kingdom [92]. The imperative role of BRs in stress management has been explored in many plants subjected to temperature, water, salt, pesticide, and heavy-metal stresses [9,10,11,24,75,93,94,95,96,97,98,99,100]. Osmolytes or compatible solutes have an important role in maintaining osmotic homeostasis as a protective mechanism during stressful conditions [101]. Reports have documented that BRs can regulate abiotic stress by influencing various types of osmoprotectants [96,102]. BR signaling controls the various physiological processes of plants under stress conditions, and BRL3 (vascular-enriched member of the BR receptor family) is thought to regulate the biosynthesis of all key osmoprotectants [103]. Overexpression of BRL3 in *Arabidopsis* under drought stress has been observed to enhance accumulation of important osmolytes, which confer stress tolerance [103]. Figure 2 explains the impact of BRs on osmolytes in plants growing under drought conditions.

### 3.1. Brassinosteroids and Proline

It is an amino acid that functions as a molecular chaperone as it aids in scavenging of free radicals as well as stabilizing the redox status of the cytosol in the cell [104,105,106]. A number of studies have shown enhanced content of proline in response to BRs. A study conducted on plants of *Raphanus sativus* subjected to Cu and Cr stress when treated with 24-epibrassinolide showed a significant increase in proline content [107,108]. Similarly, *Cucumis sativus* under Cu stress showed an enhanced content of proline when 24-epibrassinolide treatment was given [109]. Another report on *Salvia miltiorrhiza* demonstrated enhanced proline content when foliar spray of BRs was given to drought-stressed plants [110]. The treatment of 24-epibrassinolide was also found to be beneficial for *Capsicum annuum* var. *frutescens* plants that were subjected to water stress and with foliar spray, a significant increase in proline was observed [111]. The same study also proved that a synthetic brassinosteroid, 7,8-dihydro-8α-20- hydroxyecdysone, which is produced at much lower cost, was able to mimic the effects of naturally produced 24-epibrassinolide. 28-homobrassinolide was also reported to have similar response in Cu-stressed *Brassica juncea* plants [112]. Salt stress given to *Vigna radiata* plants showed enhanced proline content and combination of salt and brassinolide showed maximum accumulation of proline [113]. The accumulation of proline might be due to enhanced gene expression of biosynthetic genes [113]. The fruits of *Prunus persica* that were kept in cold storage were shown to be protected from chilling injury through changes in proline metabolism when treated with 24-epibrassinolide. The results showed that the fruits had increased proline content as well as Δ1-pyrroline-5-carboxylate synthetase (P5CS) activity, while a decrease in the activity of proline dehydrogenase was seen [114]. Glutamate becomes the precursor of proline synthesis in the presence of enzymes P5CS and Δ1-pyrroline-5-carboxylate reductase [115]. Proline dehydrogenase, however, catalyzes the degeneration of proline [116]. Apart from the effect on endogenous levels of proline, studies have also shown that exogenous application of BRs in combination with proline can be useful in alleviation of abiotic stresses. The two cultivar of *Brassica juncea* (Varuna and RH-30) grown under saline conditions when treated with proline and 24-epibrassinolide were able to resist the adverse effects of salinity and showed better photosynthetic efficiency and enhanced yield [117].

### 3.2. Brassinosteroids and Glycine Betaine

Glycine betaine belongs to the group of compatible osmolytes, the molecules of which remain unchanged at neutral pH [118,119,120,121]. Due to this, these have high solubility in water and are not present in the hydration sphere of proteins, and hence they aid in water retention and maintaining the protein structure [121]. GB accumulation in response to several abiotic stresses has been reported in many studies and has been established to possess stress protective properties. The accumulation of GB has been reported to occur maximally in chloroplasts of young leaves [19,122]. Therefore, these are the main sites of biosynthesis, and from the site of synthesis it is translocated to other parts through phloem [121,123,124]. Hence, the stress tolerance ability of GB is usually attributed to chloroplast-produced GB [119]. It aids in maintaining the photosynthetic machinery as well as the enzymes and lipids, which are essential for electron flow inside and outside the thylakoid membrane and is released in response to signals produced by stress [19,122]. The photosystem II is one of the key components of the photosynthetic machinery, which is also most susceptible to adverse effects of stress, and GB plays a vital role in maintaining its stability [118,125]. Thus, GB through photosynthetic efficiency maintains plant growth and yield [121].

Brassinosteroids, when given to plants exogenously, show a positive effect on GB accumulation, thereby aiding in stress tolerance by enhancing its levels. The seven-day-old plants of *Raphanus sativus* that were subjected to Cr stress were treated with 24-epibrassinolide and it was observed that the content of GB enhanced significantly, thereby enhancing the tolerance of the plants against Cr stress [126]. Tolerant and sensitive varieties of *Pisum sativum* plants were tested for understanding the effect of 24-epibrassinolide against salt stress. It was found that both tolerant and sensitive varieties showed enhanced content of GB in response to salinity, and when 24-epibrassinolide treatment was given, a further increase in its content was reported thereby establishing its stress protective capability [127]. Another study on salt-stressed tomato plants was conducted to understand the role of 24-epibrassinolide in improving plant’s resistance. It was found that the seeds pre-soaked in 24-epibrassinolide solution, when grown in saline conditions, showed a better accumulation of GB than the untreated seeds that were subjected salinity stress [128]. The effect of 24-epibrassinolide was also noticed on *Pisum sativum* seedlings subjected to Cd stress. It was reported that Cd stress enhanced the content of GB and the co-application of Cd and 24-epibrassinolide caused an additional increase in its levels, thus increasing the stress tolerance ability of the plants [129]. The reason for BR-mediated biosynthesis of GB is due to better performance of the key enzyme betaine aldehyde dehydrogenase (BADH), which is involved in the formation of GB from choline [94,130].

### 3.3. Brassinosteroids and Polyamines

Polyamines are aliphatic nitrogen-containing compounds that are of low molecular weights and are polycationic in nature [131]. Evidence from the studies has pointed out that polyamines have a significant role in abiotic stresses, which has been assessed by the change in the levels of polyamines in response to stress [132]. The three major polyamines *viz*. putrescine (Put), spermidine (Spd), and spermine (Spe) have been reported to undergo increase in their contents when the plants are subjected to abiotic stresses [133]. Effects of BRs on polyamines in relation to abiotic stress tolerance have come to light, which indicates some crosstalk between the two that leads to stress tolerance. It was suggested by Takahashi and Kakehi [134] that BRs are instrumental in maintaining the normal levels of spermidine, due to which normal growth of plant is maintained. Also, the same study suggested that BR application can enhance levels of Put, which aided in stress tolerance. In another study, the seven-day-old seedlings of *Raphanus sativus* when grown under Cu stress showed alteration in polyamine content. It was suggested in the study that BR application to Cu-stressed seedlings helped in stress mitigation through changes on polyamine levels [107]. Cu stress along with BR application also caused a decrease in the levels of cadaverine, which further enhanced the activity of superoxide dismutase for scavenging of ROS [135]. The interaction of polyamines and BRs was also demonstrated in another study on *R. sativus*, in which the effects of toxic concentrations of Cu were alleviated by co-application of Spd and BRs. It was also pointed out that the expression of genes involved in polyamine enzymes was modulated, which led to stress tolerance [93].

### 3.4. Brassinosteroids and Sugars/Sugar Alcohols

Out of various osmoprotectants, sugars have the maximum contribution in maintaining osmotic potential, particularly under osmotic stress [136]. During high salinity or drought conditions, the contents of various sugars usually increase even though there is reduction in CO_2_ assimilation [137]. The soluble sugars have a major role in maintenance of osmotic homeostasis as well as scavenging of ROS [136]. Sugars have been reported to have the dependency on BRs for their growth responses particularly in darkness [138]. The study conducted on *Arabidopsis* showed that brassinazole, which is a biosynthetic inhibitor of BRs, repressed the genes that regulated sugar-induced hypocotyl elongation [139]. The study also established that sugar treatments, when applied exogenously, could enhance the transcription of *BZR1* and *BES1*. These are the transcription factors that are responsible for mediating BR responses. This suggested that sugar application to plants grown in dark conditions could aid in maintaining high contents of BZR1 proteins [139].

## 4. Role of Ethylene in Regulation of Osmolytes Under Abiotic Stress

Ethylene, a gaseous phytohormone, affects seed germination, plant development, leaf and flower senescence, fruit ripening, as well as imparting abiotic stress tolerance [140]. It is a well-known stress hormone [141] that has been reported to provide tolerance towards salt, heat, drought, and ozone stress [142,143]. Ethylene has been documented to trigger expression of certain genes essential for proclaiming resistance or adaptation towards abiotic stresses [144]. Ethylene may regulate the abiotic stress tolerance through its influence on different osmolytes. There might be some correlation between ethylene synthesis and regulation of osmolyte accumulation that can protect the plants under various stressful conditions. Iqbal et al. [145] reported the involvement of ethylene in regulating abiotic stress through its impact on osmoprotectants accumulation. Studies carried out at *ein2-5* and *ein3-1* (ethylene insensitive) mutants also confirmed the involvement of ethylene in osmolyte biosynthesis [146]. Expression of *P5CS1* (pyrroline-5-carboxylate synthetase, involved in proline biosynthesis) was up-regulated in control plants under drought stress, but remained unchanged in *ein* mutants [146]. In the following section, the role of ethylene in regulating osmolytes under abiotic stress is discussed.

### 4.1. Ethylene and Proline

Proline accumulates in cytosol and protects enzymes and membranes from stress. It also stabilizes the membranes and acts as free radical scavenger under abiotic stress conditions [61]. Recently, reports have shown higher accumulation of proline in *Arabidopsis altissima* seedlings in the presence of salt and less water conditions [147]. Studies have also suggested that exogenously applied proline induces the endogenous levels of various important osmolyte that promote growth and decreases uptake of Na^+^ and Cl^−^ ions [148,149].

The synthesis of proline is related to assimilation of nitrogen [150]. The proline formation in plants mainly occurs from glutamate through pyrroline-5-carboxylate (P5C) with the help of P5C synthetase (P5CS) and P5C reductase. Mitochondrion is the site in plants where the step-wise degradation of proline occurs, catalyzed by proline dehydrogenase or proline oxidase, which forms proline from P5C. Many studies have noticed the role of ethylene in providing tolerance towards abiotic stress through its effect on N assimilation and proline accumulation [23,151]. Expression of *P5CS1* (pyrroline-5-carboxylate synthetase 1) was up-regulated in control plants under drought stress but remained unchanged in *ein* mutants [146]. Khan et al. [23] reported regulation of proline concentration in *Triticum aestivum* under heat stress. Recently, Iqbal et al. [145] showed the involvement of proline and ethylene in providing salt tolerance in *Brassica juncea*. Further, they found that nitrogen maintained the production of proline by enhancing the activity of proline-metabolizing enzymes that protects the plants by regulating ethylene level [145]. Earlier, Chrominski et al. [152] reported increased conversion of ACC into ethylene upon salt and water stress in *Allenrolfea occidentalis* and later found that exogenous application of proline balances the level of ethylene under such conditions.

### 4.2. Ethylene and Glycine Betaine

**Figure 3:**
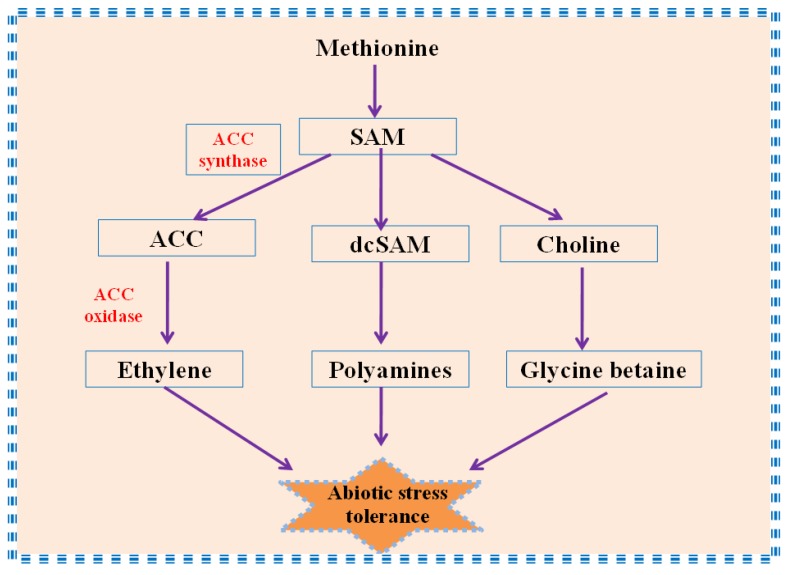
Role of ethylene, glycine betaine (GB), and polyamines under abiotic stress, after their formation through a common precursor, S-adenosylmethionine (SAM). (ACC: 1-amino-cyclo-propane-1-carboxylic acid; SAM: S-adenosyl methionine; dcSAM: SAM decarboxylase enzyme).

Glycine betaines are quaternary ammonium compounds that are key osmoprotectants and have the potential to alleviate harmful effects of different abiotic stresses. It gets accumulated in chloroplast of plants, thereby providing tolerance towards stresses [153]. Ethylene is essential in less concentration for plant growth [154]. Further, enhanced ethylene synthesis is related to plant abiotic stress responses [155]. The first step in the formation of ethylene is the formation of S-adenosylmethionine (SAM) from methionine. The SAM formed during ethylene synthesis is also a precursor for GB biosynthesis (Figure 3). Under salt stress, Khan et al. [51] reported that application of salicylic acid increased the concentration of methionine and accumulated GB in *Vigna radiata* plants. However, the formation of ethylene was reduced by inhibiting ACC synthase enzyme. Thus, the enhanced accumulation of GB and reduced ethylene under salt stress increased the glutathione (GSH) content, thereby lowering oxidative stress [51]. Moreover, in tobacco and tomato, the production of ethylene is increased in response to cold stress, whereas the plants showed no accumulation of GB [156]. However, in bean and wheat, GB gets accumulated in plants with decreased ethylene production during cold stress [157]. These strikingly inverse relations between ethylene and GB can be due to the donation of methyl group by SAM to choline, which is the only link between the two biosynthesis pathways [119] (Figure 3). Many reports have documented the role of GB and ethylene in providing tolerance towards cold stress [46,158,159] by scavenging the excess electrons produced in electron transport chains, which suggests linkage between the biosynthesis of GB and endogenous levels of ethylene [119]. The reports mentioning direct involvement of ethylene in mitigating light stress is less, however endogenous ethylene controls the synthesis of GB, which alleviates light stress-induced responses in plants [90].

### 4.3. Ethylene and Polyamines

Polyamines are widely distributed in plants and regulate different cellular functions that are important for growth of cells. Plants accumulate a large quantity of polyamines against different abiotic stresses [160]. Many researchers have reported that exogenous applied polyamines protect the plant from drought [161] and salt stress [135].

Polyamine biosynthesis is related to ethylene biosynthesis, since their precursor (S-adenosylmethionine, SAM) is common [162] (Figure 3). Reports suggest strong correlation between ethylene and polyamines in response to ROS formation in leaves of spring wheat seedlings under osmotic stress [163]. The results showed increased polyamine content in response to stressed conditions, reduced ROS production, and ethylene synthesis in plants, however, increased ROS production triggered ethylene synthesis, which promoted oxidation of polyamines and reduced its content in plants [163]. Similar results were obtained by Li and Wang [164] in leaves of *Glycyrrhiza inflata* seedlings grown under osmotic stress.

### 4.4. Ethylene and Sugars/Sugar Alcohols

Sugars provide energy for proper functioning and metabolism of plants. Sugars regulate osmotic adjustment and provide protection to membranes by scavenging ROS against different stresses [165,166]. Many studies reported accumulation of osmoprotectants such as reduced sugars like glucose, fructose, and fructans under drought and salt stress [137,167]. Trehalose, an oxidized sugar, acts as an osmolyte and protects proteins under stressful conditions [166]. Pilon-Smits et al. [168] reported tolerance against drought stress in sugar beet upon accumulation of fructan. Similarly, another sugar, trehalose, provided protection from heat and salt stress in wheat and rice plants [148,169,170].

Sugar alcohols also known as polyols are structurally divided into two types, i.e., cyclic, such as myoinositol and pinitol, and linear, which includes sorbitol, mannitol, ribitol, and xylitol. These polyols are water soluble and provide tolerance potential to plants against various abiotic stresses. Polyols regulate osmotic balance, sequester Na^+^ in the vacuole or apoplast, thus protecting membranes against drought [171] and salt stress [172]. Several studies have reported the role of sugar alcohols in abiotic stress. For example, Patonnier et al. [173] and Kaya et al. [174] suggested the role of mannitol in providing tolerance against drought and salt stress in *Fraxinus excelsior* and *Zea mays*. Similarly, D-ononitol was reported to be accumulated in *Arabidopsis thaliana* plants in response to drought and salt stress [175]. The role of ethylene in regulating sugars under abiotic stress can be correlated with scavenging ROS produced during stressful conditions. However, further research is needed to find out the mechanism involved in regulating stress tolerance in plants mediated by interaction between ethylene biosynthesis and sugars.

## 5. Role of Salicylic Acid in Regulation of Osmolytes Under Abiotic Stress

Salicylic acid (SA) is a plant phenolic hormone, ubiquitously present in plants. Recently, it has been recognized as an imperative molecule participating in local and systemic response to stress [5]. In order to combat abiotic stress-induced production of ROS, plants have developed certain mechanisms to acclimatize to ionic and osmotic stress. To maintain the osmotic potential of plant cells under stress, they have evolved a protective mechanism i.e., osmoregulation arbitrated by various osmolytes including proline, soluble sugars, amines, and GB, etc. They play a significant role in turgor-maintenance in plants under abiotic stress [176]. SA is largely reported to modulate osmolytes synthesis such as GB, proline, and sugars under abiotic stress [177,178,179].

### 5.1. Salicylic Acid and Proline

Scientists have well documented that SA is involved in enhancing synthesis of proline under abiotic stress [23]. Activity of proline biosynthetic enzymes viz. γ-glutamyl kinase and pyrroline-5-carboxylate were significantly elevated in 0.5 mM SA-treated *Lens esculenta* plants under salt stress. The elevation in metabolism of proline is attributed to a stress tolerance mechanism in salt-stressed plants [176]. Furthermore, Misra and Misra [177] suggested enhancement in proline metabolism enzymes (pyrroline-5-carboxylate and γ-glutamyl kinase) and lowered the activity of proline oxidase responsible for enhanced biosynthesis of proline in retaliation to abiotic stress. The increment in levels of proline under salt stress in *Rauvolfia serpentina* plants also advocated significant participation of SA in regulation of cell turgor. Another observation by Khan et al. [23] reported SA (0.5 mM) treatment enhanced tolerance of *Triticum aestivum* plants to heat stress by increasing the content of proline in response to SA-influenced elevation in γ-glutamyl kinase activity and decline in proline oxidase activity. Moreover, elevation in proline levels by SA application was argued to boost nitrogen assimilation and improve photosynthesis [180].

Exogenous application of proline also led to enhancement endogenous SA accumulation (influenced by NDR1-dependent signaling pathways) and resulted in regulating Ca-mediated oxidative stress as a defense response in plants [180]. SA is reported to ameliorate stress generated in response to salt stress in *Torreya grandis* seedlings through proline accumulation. Enhanced proline contents are involved in biosynthesis of stress-protective proteins, which are imperative for stress response in plants [181]. Proline levels and performance of antioxidative enzymes in *Brassica juncea* plants were enhanced after SA treatment under heat stress [182]. Another report by Ma et al. [183] reported alleviation of salinity stress in *Dianthus superbus* by elevation in activity of antioxidative defense system, photosynthetic attributes, and proline levels. Elevation in proline content contributes to maintenance of osmotic homeostasis at cellular levels and protects membrane integrity. Enhanced accumulation of proline after SA application is due to the regulation of expression patterns of genes involved in proline biosynthesis. Key genes like *P5CSA* and *P5CSB* (encoding pyrroline-5-carboxylate synthase) gets up-regulated, whereas *PDH* (encoding proline dehydrogenase) gets down-regulated after SA application [184].

### 5.2. Salicylic Acid and Glycine Betaine

SA and analogs of aspirin induces GB synthesis in the range of 0.5–2.5 mM in plants exposed to salinity, water deficit, and chilling stress [185]. Salicylates and aspirin-influenced enhancement in GB accumulation is an imperative part of systemic resistance in stressed plants. GB also affects the activity of protein kinases under hyperosmotic stress [186]. SA-influenced increase in GB content has been reported to enhance the overall growth of plants [177]. Similarly, alleviation of salinity stress in *Vigna radiata* plants by SA involves GB accumulation. SA (0.5 mM) treatment increased GB content by increasing methionine level as well as suppression of ethylene production under salt stress. Similar effects were observed by the same co-workers after exogenous application of SA analogs i.e., 2, 6, dichloro-isonicotinic acid on GB content, which aids in amelioration of salt stress-influenced effects on growth and photosynthesis. Another report by Aldesuquy et al. [187] suggested that co-application of GB (10 mM) and SA (0.05 M) exogenously to two cultivars (sensitive Sakha 94 and resistant Sakha 93) of *Triticum aestivum* had a repairing effect on growth and metabolism of plants grown under water deficit conditions. Similar effects of simultaneous application of GB and SA was found to lower the drastic effect of drought stress in *Helianthus annuus* plants [188].

### 5.3. Salicylic Acid and Polyamines/other Amino Acids

Salicylic acid triggers the accumulation of proline in plants under stress conditions. Enhanced accumulation of polyamine is due to the up-regulation of the transcript levels of key genes like *SPMS* (spermine synthase) and *SPDS* (spermidine synthase) are involved in polyamine biosynthetic pathways [189]. Moreover, genes like *SAMS* (S-adenosyl-l-methionine synthase) and *SAMDC* (S-adenosyl-l-methionine decarboxylase), which are involved in SAM biosynthesis (precursor of polyamines), are also up-regulated by SA [189]. Exposure of aging to leaf tissue excised from plants resulted in alteration of metabolic activities, such as stimulation of de-novo synthesis of amino acids modulated by SA application in response to regulation of redox status of stressed plants and defense response [190]. Earlier studies also suggest an imperative role of SA in alteration of amino acid balance in stressed plants revealed by studies on homoserine kinase and amino transferases [191]. Various amino acids, including methionine and aromatic amino acids viz. tyrosine and phenylalanine, were elevated by exogenous application of SA (1 mM) in *Glycine max* plants exposed to salinity stress [192]. They further suggested that these amino acids act as a sink of excess nitrogen in plants under salt stress and under non-saline conditions.

The free amino acids have imperative participation in regulating osmotic homeostasis in plant tissue [193]. Similarly, Aldesuquy et al. [187] suggested elevation in free amino acid content resulting in enhancement in proteolytic activities in response to altered osmotic status of plant cells. The increased levels of free amino acids in plants during the whole life cycle also revealed possible participation of these compounds in osmoregulation [194]. Another report by Yadav et al. [194] and Sankar et al. [195] revealed elevation in amino acid levels of *Sorghum bicolor* and *Abelmoschus esculentus* under water deficit conditions. Another observation by El-Tayeb et al. [196] suggested up-regulation in amino acid levels in *Helianthus annuus* plants by SA application under Cu metal stress. Also, Hussein et al. [197] revealed similar elevation in proline and GB content in *Zea mays* plants exposed to salinity stress by exogenous SA application. Another report by Abd Allah et al. [198] showed similar enhancement in amino acid content in quinoa plants by exogenous application of SA at different concentrations i.e., 200 and 400 mg L^−1^ under drought stress. They further suggested elevation in amino acid content under water deficit conditions might be attributed to enhanced protein degradation.

### 5.4. Salicylic Acid and Sugar/Sugar Alcohols

An evidently solid association occurs between sugar levels and osmotic stress and has been supported by transgenic studies [22]. Exogenous application of SA (10^−2^ M) to *Zea mays* plants exposed to salinity stress resulted in enhancement in content of soluble sugars [199]. SA supplementation might result in activation of metabolic pathways, which consume soluble sugars that consequently result in the replenishment of growth of stressed plants [200]. Furthermore, SA treatment is assumed to inhibit the activity of polysaccharide hydrolyzing enzymes and accelerate soluble sugar content [199]. Total sugars and carbohydrate levels were reported to be elevated in quinoa plants by exogenous application of SA [198]. They further revealed that they play an important part in raising plants under osmotic stress, stabilizing protein, and membrane integrity. According to Bohnert and Jensen [201], carbohydrates contribute as ROS scavengers and membrane stabilizer. The substantial enhancement in carbohydrate levels might also be due to the recovery of photosynthetic apparatus of stressed plants. SA application is also suggested to inhibit polysaccharides hydrolyzing enzymes [202]. Table 1 summarizes reports of SA-influenced alterations in osmolyte content in plants exposed to abiotic stresses.

## 6. Role of Cytokinins in Regulation of Osmolytes under Abiotic Stress

Phytohormone cytokinins control large number of activities in plants, which are important to their growth and development [219,220]. In tissue culture, cytokinins stimulate the process of cell division and act as a main regulator of cell cycle [221]. In combination with auxin, it plays a significant role in controlling cell divisions and maintaining the stem cells of root and shoots apical meristems [222,223]. Other major functions of cytokinins are, including but not limited to, reduction in the initiation of lateral roots [224], control of cell division, phloem and metaxylem differentiation in roots [225], morphogenic differentiation during sunlight in spreading leaves [226], and decrease in senescence of leaf [227,228]. Nonetheless, cytokinins play both positive and negative roles for providing stress tolerance. According to various reports, concentration of cytokinins is found to be inhibited during enhanced stressed conditions [229]. On the other hand, some observations indicate that the level of cytokinins enhanced during stress conditions, whether it was for short or long duration [230]. Under stress conditions, regulation of key part of ethylene cell signaling, i.e., MAPK6 is considered to play an important role in biosynthesis and accumulation of osmolytes in plants [231].

### 6.1. Cytokinins and Proline

Proline is present in higher plants and during stress conditions like salinity or drought stress, its accumulation is found to enhance drastically [70]. As mentioned in earlier sections, glutamate is a precursor of proline and with the help of P5CS, it is converted to glutamate semialdehyde. Pyrroline-5-carboxylate synthetase acts as the main enzyme for the synthesis of proline, and further, glutamate semialdehyde instinctively transformed to pyrroline-5-carboxylate (P5C) [232]. P5C reductase (P5CR) causes reduction of P5C intermediate to proline. Under stress conditions, accumulation of proline acts as a sink for excess reductants, which provides NAD^+^ and NADP^+^ required for regulating respiration and photosynthesis. Proline acts as a molecular chaperone and they have the potential to maintain the protein integrity and activities of enzymes. Apart from this, it also prevents the aggregation of proteins and provides stability of M4 lactate dehydrogenase when plants are exposed to high temperatures [233]. During osmotic and heavy-metal stress, proline provides stability to nitrate reductase, ribonucleases, and proteases activities [234]. Exogenous application of cytokinin along with NaCl provides signals that go across a common pathway, which cause accumulation of PEP carboxylase and proline. Reports suggest that accumulation of PEP carboxylase and proline is triggered by cytokinin in *M. crystallinum* [235]. Addition of NaCl and 6-BAP also increase the proline accumulation. In the same way, cytokinin along with NaCl contributes in increasing PEP carboxylase and proline accumulation.

### 6.2. Cytokinins and Glycine Betaine

**Figure 4:**
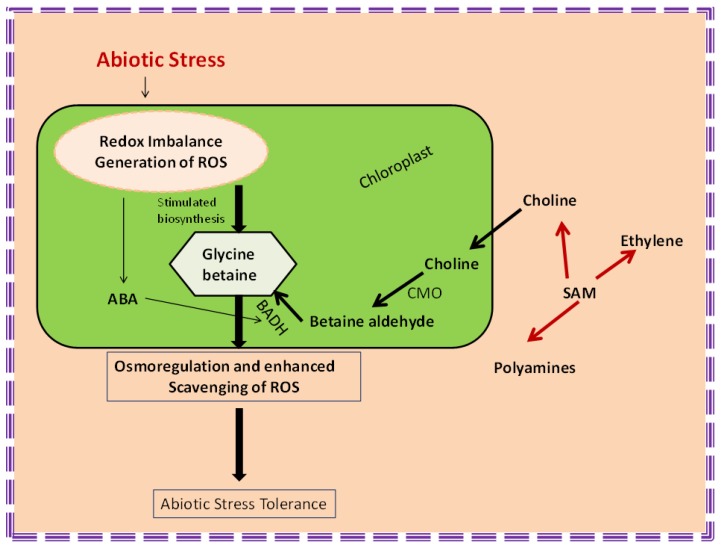
Biosynthetic pathway of GB and its role in abiotic stress tolerance (modified after Kurepin et al. [119]. BADH (betaine aldehyde dehydrogenase), CMO (choline monooxygenase), SAM (S-adenosylmethionine).

Many organisms like algae, fungi, bacteria, cyanobacteria, and animals help in accumulation of GB [236]. During various abiotic stress conditions, GB majorly accumulates quaternary ammonium [237]. Choline is the precursor of GB and by the action of choline monooxygenase (CMO) and BADH, transformed into betaine aldehyde and GB, respectively [238]. Accumulation of GB stabilizes the structure of macromolecules, protects the cytoplasm from ion toxicity, and stabilizes photosystem II from high temperature stress [239]. Due to extreme stress condition, PSII excitation pressure PSII [240] also increases, which further leads to redox imbalance in photosynthetic electron transport system because of ROS over accumulation. Biosynthetic pathway of GB has a common precursor (SAM) with the biosynthetic pathways of ethylene and polyamine. Increased excitation pressure causes enhanced production of photosynthetic electrons that can be used in the oxidation of choline to BA [241] and this activity is controlled by CMO (Figure 4). An increased level of GB causes reduction in redox imbalance and excitation pressure and, thus, provides tolerance against stress [119]. Enhanced endogenous level of ABA can stimulate the biosynthesis of GB from BA, and BADH regulates this process. Interactions of GB with other phytohormones provide tolerance to plants against various types of abiotic stresses [119]. It is suggested that cytokinins might regulate GB biosynthesis by modulation of the above-mentioned pathway, which needs further detailed studies. Up-regulation in the expression pattern of *CKX1* gene (cytokinin dehydrogenase1, which is responsible for the deactivation of active cytokinins) is also related to enhancement of GB levels [74].

### 6.3. Cytokinins and Polyamines

Cytokinins are important plant hormones that trigger different physiological and biochemical processes in plants either in normal or stressful environments. It is very important to note that endogenous levels of cytokinins are good indicators of stress responses, which greatly change with the severity of stress. For instance, concentration and transport of trans-zeatin riboside decreased the ABA concentration, which drastically increased in drought stress [242,243]. Interestingly, some studies show that cytokinins and polyamines mutually regulate several physiological and biochemical processes in plants showing strong correlation between the levels of cytokinins and polyamines and act as inter and intracellular messenger regulating biotic and abiotic stresses [244,245]. However, it is still unclear how polyamines regulate cytokinin signaling in plants. Although some studies showed that cytokinins increased the activities of arginine decarboxylase (ADC) and Put in cultured excised cucumber cotyledons, rice embryos, and etiolated pea seedlings [246,247,248,249]. Furthermore, etiolated cotyledon treated with kinetin boosted the activity of polyamine oxidase (PAO) whereas it decreased the activity of S-adenosylmethioinine decarboxylase (SAMDC) along with reduced levels of Spd and increased Put contents [250], indicating that polyamines may play a crucial role in regulation of gene expression cytokinins-treated cucumber cotyledons, fostering further studies that could help in establishing if there is any connection between Spm and Spd with cytokinin signaling.

### 6.4. Cytokinins and Sugars/Sugar Alcohols

During abiotic stresses, accumulation of sugars like sucrose and trehalose have enhanced in plants [251]. These sugars contribute to stabilizing the membranes, osmotic adjustment, and various other significant functions in plants during abiotic stress conditions [237]. Sugars not only contribute in growth, but also help in the regulation of the gene expression, which control respiration, photosynthesis, production, and degradation of starch and sucrose [252]. During cellular dehydration, for the establishment of hydrogen bonds, polyol hydroxyl groups efficiently replace water and provide protection to membrane structures and regulate the activities of enzymes [253].

A non-reducing disaccharide, trehalose, is soluble in nature. It is not reactive chemically, thus, compatible with cellular metabolism during abiotic stress conditions. It is found in organisms like bacteria and fungi in high concentrations [254]. In plants, during extreme dry conditions, trehalose provides stability by resurrection in halophytes and stabilizes the proteins and cell membranes [255]. It is also considered as a replacement for water because of its physical properties of maintaining hydration and, hence, contributes to the regulation of levels of macromolecules. Similarly, polyols also provide osmotic adjustment and regulate redox, while mechanisms such as fructose-6-phosphate gives rise to mannitol (also acts as osmoprotectant) by consequent action of phosphomannose isomerase, mannose-6-phosphate reductase, and mannose-1-phosphate phosphatase [256]. Hence, osmolytes contribute to the amelioration of free radicals and provide stability to structures of macromolecules [257].

## 7. Role of Jasmonates in Regulation of Osmolytes under Abiotic Stress

Among different phytohormones, jasmonates (JAs) are considered as one of the important phytohormones that have a significant role in abiotic stress tolerance. Jasmonic acid (JA) regulates different plant responses including gene regulation, biosynthesis of special proteins, as well as secondary metabolism under a broad spectrum of stressful conditions. Role of JA in growth and development of plants is well recognized in both biotic and abiotic stress conditions [4,258]. Therefore, increase in endogenous levels of JAs indicates its importance in abiotic stress tolerance, whereas exogenous application of JA provides substantial growth improvement in plants under different abiotic stresses. It is imperative to understand the mechanism of JA in up-regulating the genes contributing in osmolyte biosynthesis. In this chapter, we have summarized the beneficial role of JA in improving plant growth and development thorough its interaction with different osmolytes, specifically proline, polyamines, GB, and sugar/carbohydrates alcohols.

Jasmonates’ involvement in plant growth and development is not in a simple pathway, but rather has multifarious interconnections among different growth regulators such as ethylene, ABA, and SA [259]. After its biosynthesis, JAs interact with different amino acids to produce active compounds such as isoleucine to form isoleucine-JA or methyl jasmonate (Me-JA), which is the most active form of JAs [260]. JAs have multifaced involvement in plant stress physiology due to their role in attenuating different abiotic stresses. JAs regulate detrimental effects of these environmental factors through a cascade of plant responses. For instance, JA increased the levels of non-enzymatic antioxidants such as proline, which have been reported in numerous studies [261,262]. Studies also show that endogenously as well as exogenously applied JAs improve plant growth due to their effect on plant metabolites. Moreover, changes in the phytohormone contents in plant cells have been reported to be associated with specific gene expression, which are involved in the biosynthesis of JAs [259]. Therefore, Jas-induced abiotic stress tolerance has been considered as a promising approach carried out under numerous stresses such as salt, drought, heavy metals, pesticides, as well as light and temperature stress [4,263,264]. In the following subsections, we have summarized some of important roles JAs and its interaction with osmoprotectants such as proline, GB, polyamines, and sugars/carbohydrate alcohols.

### 7.1. Jasmonates and Proline

Proline is an important non-enzymatic osmolyte, and the level of proline under any stress indicates the ability of a plant to regulate plant protection against certain reactive oxygen species. Higher levels of proline usually enhance the plant defense while low levels indicate the lesser. JA has potential to regulate various abiotic stresses by regulating water potential in plant cells. Proline acts as an osmolyte and improves osmotic adjustment by stabilizing macromolecules by protecting them against the severities of ROS [265]. Anjum et al. [261] and Shan et al. [262] described ameliorative effects of JA in alleviating drought stress through the biosynthesis of osmolytes such as proline. JA up-regulates several key genes playing important roles in drought adaptation via stimulating various secondary metabolites, cell wall formation, and encoding stress responsive proteins and solutes such as proline [266]. JA-induced improvement in proline contents has been reported in several studies such as drought stress [267], heavy-metal toxicity [268,269,270,271,272], salt stress [273], and UV-B radiation [274,275]. Therefore, increased proline contents are good indicators of stress tolerance in plants due to their role in the reconstruction of chlorophylls and activation of Kreb’s cycle [64,276,277,278]. Exogenously applied JA also enhances the accumulation of organics acids of Kreb’s cycle *viz.* citrate, succinate, fumarate, and malate, which ultimately provide resistance against abiotic stress [4].

### 7.2. Jasmonates and Glycine Betaine

Glycine Betaine is accumulated in many organisms like algae, fungi, bacteria, cyanobacteria, and animals, helping plants to cope abiotic stresses [236]. GB is an efficient compatible solute with different roles in inducing abiotic stress tolerance in plants. It also protects the structure of vital proteins under abiotic stress conditions [279]. Literature shows that higher levels of GB causes reduction in the redox imbalance and excitation pressure and, thus, provides tolerance against different stresses [119]. Moreover, studies also suggest that a higher endogenous level of ABA may have a stimulating effect accelerating the biosynthesis of GB from BA whereas betaine aldehyde dehydrogenase enzyme regulates this process. Interactions of GB with other phytohormones provide tolerance to plants against various types of abiotic stresses [119]. In a study, application of JA increased the GB contents by 45.37% under Ni stress and improved overall plant growth in soybean seedlings [270]. Similarly, Ahmad et al. [271,280] established that JA application improved plant growth due to increased GB contents in *Solanum lycopersicum* under salt and cadmium stress.

### 7.3. Jasmonates and Polyamines

Polyamines (PAs) are important low molecular weight compounds that contribute a significant role in abiotic stress tolerance in plants. PAs are synthesized endogenously under an array of biotic and abiotic stresses and modulate adverse effects of these environmental factors by protecting membranes of vital organelles. In addition, PAs help plants in scavenging ROS and regulate plants responses to several abiotic stresses [281,282,283]. It has been shown that JAs signaling has a synergistic effect on PAs biosynthesis under stressed conditions. For instance, Spd content increased in barley genotypes and protected membranes from peroxidation due to their prime role in activating key antioxidant enzymes [267]. In another study, JA application alleviated UV-B radiation by producing important phenylpropanoid compounds including polyamines. Moreover, JA modulates the adverse effect of UV-B by mediating jasmonate inducible transcription factor in tobacco plants (*Nicotiana tabacum*) [284]. Therefore, the role of JA-induced abiotic stress tolerance is imperative to include in managing and improving plant defensive system to increase the net productivity by reducing the losses due to these environmental severities.

### 7.4. Jasmonates and Sugars/Sugar Alcohols

Sugar accumulation and osmotic stress under abiotic stress conditions show strong correlation, resulting in enhanced stress tolerance. According to Bohnert and Jensen [201], carbohydrates contribute as ROS scavengers and membrane stabilizers. The substantial enhancement in carbohydrate levels might also be due to the activation of photosynthetic machinery of stressed plants. In a recent study, researchers found that the application of methyl jasmonate improved the contents of different carbohydrate metabolites such as benzyl alcohol, phenyalacetaldehyde, benzaldehyde, 2-phenylethyl alcohol, and trans-2-hexanal in tea plants (*Camellia sinensis*) [285]. Ahmadi et al. [286] found that exogenously applied methyl jasmonate under salt stress modulated the salinity induced peroxidation through increased levels of soluble sugar in *Brassica napus* leaves. Sugars/sugar alcohols act as osmolytes and osmoprotectants and improve relative water contents under abiotic stressed conditions. Similarly, Sirhindi et al. [270] revealed that application of JA modulates the heavy metal toxicity through increased contents of proline, GB as well as soluble sugars. It is believed that higher levels of sugars accumulate due to excessive resistance of photosynthetic organelles and lesser transport of starch takes place in mesophyll cells. Some other studies also indicate that sugar levels may increase due to starch degradation under any kind of stresses [287]. JA application has been shown to increase sugar contents in different crop plants such as *Triticum aestivum*, *Brassica napus*, and *Ipoema batata* by improving overall plant performance under abiotic stresses [288,289,290].

## 8. Role of Abscisic Acid in Regulation of Osmolytes under Abiotic Stress

Abscisic acid is an important plant hormone which plays crucial role in the growth and development of plants [291,292,293,294,295]. In addition to its functions in regulating normal physiology of plants, ABA is also involved in modulation of various key physiological processes during extreme environmental conditions [296,297,298]. One of the main ABA mediated physiological process under stress conditions is the regulation of osmolytes in stressed plant cells and maintaining the osmotic adjustments [299,300,301]. Osmoprotection to stressed cells is enhanced by the ABA regulated accumulation and biosynthesis of osmolytes [296,302,303]. ABA acts as a signaling molecule to regulate the biosynthetic pathways of osmolytes at molecular level [298,302]. In the further text, we will discuss about various mechanisms modulated by ABA to regulate osmolyte accumulation.

### 8.1. Abscisic Acid and Proline

Abscisic acid plays an important role in the regulation of proline metabolism in plants facing various abiotic stresses [304,305,306]. Generally, ABA enhances the biosynthesis and accumulation of proline in plant cells to protect them from ill effects of stressful conditions [299,303,307]. The increased levels of this osmoprotectant by ABA stimulation further aid in reduction of oxidative stress [300,305]. The reason behind this increased proline accumulation is that ABA up-regulates the transcript levels of the genes encoding key enzymes of proline biosynthetic pathways like *P5CS* [298] which can be one of the possible reason behind increasing performance of proline biosynthetic pathway under abiotic stress. In *Medicago truncatula*, it was observed that the proline biosynthesis under drought stress was controlled by ABA signals [308]. ABA signaling is one of the main factors involved in controlling the proline biosynthesis in plants growing under abiotic stress conditions [302,309]. Table 2 summarizes the effect of ABA on proline accumulation under abiotic stress.

### 8.2. Abscisic Acid and Glycine Betaine

Abscisic acid stimulates the biosynthetic pathway of GB and leads to more accumulation of this osmolyte in plant cells, which ultimately helps in protection against abiotic stress [301,310]. The increased GB biosynthesis after ABA treatment under abiotic stress is due to the ABA-mediated activation of hey GB-biosynthetic enzyme BADH [301,311]. These researchers further confirmed the role of ABA in BADH up-regulation by studying drought stressed plants after fluridone treatment (an inhibitor of ABA biosynthesis). It was observed that after fluridone treatment, BADH activity was reduced, conforming positive role of ABA in BADH stimulation [301]. In a literature review by Hashemi et al. [312], it is mentioned that ABA and MAPK signaling pathways regulate the activity of BADH by modulating the phosphorylation process of important receptors like SnRK2 (belonging to sub-family of sucrose non-fermenting 1-related protein kinase 2). Moreover, overexpression of *SpBADH* isolated from a halophyte (*Sesuvium portulacastrum*) provides stress tolerance in genetically modified *Arabidopsis,* and the expression of this GB biosynthetic gene (*SpBADH*) was also up-regulated by ABA [311]. Table 2 provides some additional information about the role of ABA on GB in plants growing under challenging environments.

### 8.3. Abscisic Acid and Polyamines

Polyamines are well known to protect plant cells from abiotic stress and their levels are elevated in stressed plants by ABA [313,314]. Increased polyamine levels in stressed plant cells is due to the stimulation genes encoding various polyamine biosynthetic enzymes like spermidine synthase [315]. Rakitin et al. [316] suggested that ABA and ethylene modulate the biosynthetic pathways of each other and this is important for the regulation of polyamine biosynthesis in plants under stress conditions. Moreover, ABA also regulates the important transcriptional mechanisms involved in the biosynthesis of polyamines [317]. In drought conditions, transcript levels of *ADC2* (arginine decarboxylase2), *SPDS1* (spermidine synthase 1) and *SPMS* (spermine synthase) genes were observed to be up-regulated [317]. However, in ABA-deficient (*aba2-3*) and ABA-insensitive (*abi1-1*) mutant plants, it was noticed that transcript levels for polyamine biosynthetic genes were not up-regulated, indicating a clear role of ABA in polyamine biosynthesis [317]. In *Zea mays*, ABA-deficient mutants (*vp5/vp5*) were noticed to have reduced levels of endogenous ABA accompanied by less polyamine accumulation under salt stress. However, enhanced endogenous ABA levels under drought stress (in normal plants) were accompanied by enhanced polyamine contents [318]. So, it is also believed that stresses like drought induces the biosynthesis of ABA, which then acts as a signaling molecule to regulate the biosynthesis of polyamines [318]. Other studies also confirmed the role of ABA in polyamine accumulation as ABA treatment was noticed increase the mRNA levels of key genes including *ADC2* and *SPMS* [319,320]. Table 2 provides some additional information regarding ABA mediated accumulation of polyamines in plants growing under stress conditions.

### 8.4. Abscisic Acid and Sugars/Sugar Alcohols

Sugars are one of the most important osmolyte which protects plant cells from oxidative damage. Abscisic acid promotes the biosynthesis of sugars in plants under abiotic stress conditions and helps in improving plant’s tolerance against abnormal growth conditions [297,299,304,321]. Sugar related compounds which act as osmoprotectants like thiols and trehalose are also get accumulated in plants cells to regulate the cellular osmotic adjustments under stress conditions [300,304]. ABA signaling is also involved in regulation of sugar metabolism in plants [322]. Glucose and fructose levels get enhanced during water deficit conditions, accompanied by enhanced vacuolar invertase activity, and gene expression of invertase enzyme (*IVR2*) is enhanced by ABA [323,324,325]. It is mentioned by Farooq and Bano [326] that ABA regulates soluble sugar accumulation by modulating the activity of amylase enzyme under stress conditions. Table 2 summarizes some reports explaining effect of ABA on sugar accumulation in plants under different stresses.

## 9. Conclusions

Plant hormones trigger the biosynthesis of osmolytes in plants growing under challenging environmental conditions. These compatible solutes maintain the redox balance of cells by enhancing the efficiency of ROS scavenging and result in reduction of oxidative damage to plant cells. The identification and characterization of key genes involved in phytohormone-mediated stimulation of osmolyte biosynthetic pathways will give new direction to establish stress-resistant crop varieties.

## Figures and Tables

**Table 1:** SA-influenced alterations in osmolyte content in plants exposed to abiotic stresses.

Plant Species	SA conc.	Abiotic Stress	Response of plants	Reference
*Brassica juncea*	1 mM	Heavy metal (Pb- 0.25, 0.50, and 0.75 mM)	Content of proline, trehalose, and GB were increased in response to SA treatment	[96]
	1 mM	Heavy metal (Pb-0.25, 0.50, and 0.75 mM)	Levels of total carbohydrates and reducing sugar were enhanced significantly	[75]
	0.5 mM	Water deficit	Activity of enzymes including γ-glutamyl kinase and proline oxidase were enhanced	[203]
	0.5 mM	Salinity (100 mM)	Content of glutathione, an essential amino acid, was elevated	[204]
	10^−5^ M	High Temperature	Levels of proline were significantly augmented	[182]
*Cucumis melo*	0.01, 0.05, 0.1 and 0.2 mM	Heavy Metal (Cd-50, 200, 400, and 800 µM)	Significant elevation in level of proline was observed by SA pre-treatment	[205]
*Dianthus superbus*	0.5 mM	Salinity (0.3%, 0.6%, and 0.9%)	Proline content was enhanced by 53.7% under 0.3% salinity and 54.1% under 0.6% salinity	[183]
*Eleucine coraccana*	0.2 mM	Heavy Metal (Ni-0.5 mM)	Level of proline was lowered in roots and shoots both	[206]
*Glycine max*	1 mM	Salinity (4, 7, and 10 ds/m)	Lysine, methionine, isoleucine, and leucine contents were significantly enhanced. Phenylalanine and threonine levels were not influenced. Contents of non-essential amino acids *viz.* alanine, aspartic acid, glutamic acid, glycine, and serine were also augmented	[192]
	100, 200, 300 and 400 µM	Heavy Metal (As-10, 25, 50, 75, 100, and 125 µM)	Remarkable enhancement in proline levels was observed by SA dose	[207]
*Lens esculenta*	0.5 mM	Salinity (100 mM)	Activity of proline biosynthetic enzymes viz. γ-glutamyl kinase and pyrroline-5-carboxylate were significantly elevated. Similar enhancement in GB content was recorded.	[176]
*Matricaria chamomilla*	1, 10, 25 and 100 mg	High Temperature	Free proline concentration was significantly increased	[208]
*Medicago sativa*	0.2 mM	Heavy Metal (Mg-10 µM)	Proline levels were enhanced	[209]
*Pisum sativum*	2 mM	Heavy Metal (Cd-0.75 and 1.5 mM)	Level of proline was further enhanced by SA supplementation	[210]
*Solanum lycopersicum*	0.01 mM	Salinity (100 mM)	Soluble sugar and proline content were enhanced significantly	[211]
*Torreya grandis*	0.5 mM	Salinity (0.2% and 0.4%)	Augmentation in proline content was observed in response to SA application	[181]
*Triticum aestivum*	10^−4^ M	High Temperature	Proline level was elevated by 21% and sugar accumulation by 81% in field studies	[212]
	1.44 and 2.88 mM	Water deficit	Proline and GB content were increased in response to SA treatment	[213]
	1 mM	Heavy Metal (As-50, 100, 150, 200, 250, 300, 350, and 400 µM)	Proline content was elevated by treatment of As stressed plants with SA	[214]
	0.5 mM	High Temperature	Enhancement in proline content was observed	[23]
	0.05 M	Water Deficit	Soluble sugars i.e., glucose, sucrose and total soluble sugars were elevated. Polysaccharides and total carbohydrate content were also enhanced. Elevation in glutamic acid, aspartic acid, leucine, tyrosine, alanine, and isoleucine etc., were elevated	[187]
	100 ppm	Heavy Metal (Cu-5, 10, 20, and 40 mg L^−1^)	Soluble and total carbohydrates levels were enhanced by SA supplementation. Similar elevation in contents of proline and amino acid was observed	[215]
*Zea mays*	100 µM	High temperature	Proline, GB, and trehalose accumulation were increased	[216]
	500 µM	Heavy Metal (Cd-100 µM)	Total soluble sugar and proline levels were enhanced by SA application.	[217]
	500 µM	Heavy Metal (Cd-10, 15, and 25 µM)	SA counter Cd stress by enhancing the levels of proline endogenously	[218]
	10^−2^ M	Salinity (50, 100, and 150 mM)	Soluble sugar levels and polysaccharide content were recorded to be lowered	[199]

**Table 2:** Summary of the effects of abscisic acid on osmolytes under various abiotic stresses.

Plant Species	Stress	Impact on Osmolytes	Reference
*Atriplex halimus*	Salt	Increase in the contents of proline, glycine betaine, putrescine (Put), spermidine (Spd) and spermine (Spe).	[296]
*Brassica napus*	Cold	Increase in the contents of proline and soluble sugars.	[310]
*Cicer arietinum*	Heat	Increase in the contents of osmoprotectants like proline, glycine betaine and trehalose, accompanied by improved plant tolerance	[304]
	Cold	Increase in the content of proline.	[327]
*Cynodon dactylon*	Cold	Increase in the contents of proline accompanied by improvement in antioxidative response.	[328]
*Glycine max*	Drought	Increase in content of Put, but Spe and Spd contents were decreased.	[314]
*Medicago sativa*	Drought	Increase in proline content of shoots.	[329]
*Oryza sativa*	Salt	Increased accumulation of proline accompanied by regulation of expression levels of genes encoding proline biosynthetic enzymes (*P5CS* and *P5CR*).	[298]
	Drought	Increased accumulation of total soluble sugars.	[297]
	Salt	Increase in the contents of proline and soluble sugars.	[321]
*Phaseolus vulgaris*	Salt	Increase in the contents of Put, Spd and Spe.	[307]
*Phragmites communis*	Heat	Increase in the contents of proline and total thiols accompanied by improvement in antioxidative response.	[300]
*Triticum aestivum*	Drought	Increase in the contents of proline, glycine betaine and total soluble sugars accompanied by reduction in oxidative stress.	[303]
	Drought	ABA signaling regulated the metabolism of osmolytes like proline and polyamines.	[302]
	Drought	Contents of proline and soluble carbohydrates were increased, but increase was not significant.	[306]
*Ulmus minor*	Drought	Increase in proline content.	[330]
*Vigna radiata*	Drought	Increase in proline and sugar contents.	[326]
*Vitis vinifera*	Drought	Increase in the contents of Put, Spd and Spe	[313]
	Cold	Increase in the contents of proline and soluble carbohydrates.	[299]
*Zea mays*	Drought	Increase in glycine betaine content accompanied by enhanced activity of betaine aldehyde dehydrogenase (BADH).	[301]
	Salt	Endogenous ABA stimulated biosynthesis of polyamines.	[318]

## References

[1] Hussain S., Khaliq A., Matloob A., Wahid M.A., Afzal I. (2013). Germination and growth response of three wheat cultivars to NaCl salinity. Soil Environ..

[2] Saud S., Chen Y., Long B., Fahad S., Sadiq A. (2013). The different impact on the growth of cool season turf grass under the various conditions on salinity and draught stress. Int. J. Agric. Sci. Res..

[3] Sharma A., Yuan H., Kumar V., Ramakrishnan M., Kohli S.K., Kaur R., Thukral A.K., Bhardwaj R., Zheng B. (2019). Castasterone attenuates insecticide induced phytotoxicity in mustard. Ecotoxicol. Environ. Saf..

[4] Sharma A., Kumar V., Yuan H., Kanwar M.K., Bhardwaj R., Thukral A.K., Zheng B. (2018). Jasmonic Acid Seed Treatment Stimulates Insecticide Detoxification in *Brassica juncea* L.. Front. Plant Sci..

[5] Khan M.I.R., Fatma M., Per T.S., Anjum N.A., Khan N.A. (2015). Salicylic acid-induced abiotic stress tolerance and underlying mechanisms in plants. Front. Plant Sci..

[6] Anjum S.A., Tanveer M., Hussain S., Bao M., Wang L., Khan I., Ullah E., Tung S.A., Samad R.A., Shahzad B. (2015). Cadmium toxicity in Maize (*Zea mays* L.): Consequences on antioxidative systems, reactive oxygen species and cadmium accumulation. Environ. Sci. Pollut. Res..

[7] Shahzad B., Tanveer M., Hassan W., Shah A.N., Anjum S.A., Cheema S.A., Ali I. (2016). Lithium toxicity in plants: Reasons, mechanisms and remediation possibilities-A review. Plant Physiol. Biochem..

[8] Shahzad B., Tanveer M., Rehman A., Cheema S.A., Fahad S., Rehman S., Sharma A. (2018). Nickel; whether toxic or essential for plants and environment-A review. Plant Physiol. Biochem..

[9] Tanveer M., Shahzad B., Sharma A., Khan E.A. (2019). 24-Epibrassinolide application in plants: An implication for improving drought stress tolerance in plants. Plant Physiol. Biochem..

[10] Tanveer M., Shahzad B., Sharma A., Biju S., Bhardwaj R. (2018). 24-Epibrassinolide; an active brassinolide and its role in salt stress tolerance in plants: A review. Plant Physiol. Biochem..

[11] Shahzad B., Tanveer M., Che Z., Rehman A., Cheema S.A., Sharma A., Song H., Rehman S.U., Zhaorong D. (2018). Role of 24-epibrassinolide (EBL) in mediating heavy metal and pesticide induced oxidative stress in plants: A review. Ecotoxicol. Environ. Saf..

[12] Bray E.A., Bailey-Serres J.W., Weretilnyk E., Gruissem W., Jones R. (2000). Responses to abiotic stresses. Biochemistry and Molecular Biology of Plants.

[13] Shafi M., Bakht J., Hassan M.J., Raziuddin M., Zhang G. (2009). Effect of cadmium and salinity stresses on growth and antioxidant enzyme activities of wheat (*Triticum aestivum* L.). Bull. Environ. Contam. Toxicol..

[14] Khan M.I.R., Iqbal N., Masood A., Khan N.A. (2012). Variation in Salt Tolerance of Wheat Cultivars: Role of Glycinebetaine and Ethylene. Pedosphere.

[15] Sharma A., Thakur S., Kumar V., Kanwar M.K., Kesavan A.K., Thukral A.K., Bhardwaj R., Alam P., Ahmad P. (2016). Pre-sowing Seed Treatment with 24-Epibrassinolide Ameliorates Pesticide Stress in *Brassica juncea* L. through the Modulation of Stress Markers. Front. Plant Sci..

[16] Bohnert H.J., Nelson D.E., Jensen R.G. (1995). Adaptations to Environmental Stresses. Plant Cell.

[17] Sharma A., Shahzad B., Rehman A., Bhardwaj R., Landi M., Zheng B. (2019). Response of Phenylpropanoid Pathway and the Role of Polyphenols in Plants under Abiotic Stress. Molecules.

[18] Giri J. (2011). Glycinebetaine and abiotic stress tolerance in plants. Plant Signal. Behav..

[19] Chen T.H., Murata N. (2002). Enhancement of tolerance of abiotic stress by metabolic engineering of betaines and other compatible solutes. Curr. Opin. Plant Biol..

[20] Anjum S.A., Ashraf U., Tanveer M., Khan I., Hussain S., Shahzad B., Zohaib A., Abbas F., Saleem M.F., Ali I. (2017). Drought Induced Changes in Growth, Osmolyte Accumulation and Antioxidant Metabolism of Three Maize Hybrids. Front. Plant Sci..

[21] Anjum S.A., Tanveer M., Hussain S., Shahzad B., Ashraf U., Fahad S., Hassan W., Jan S., Khan I., Saleem M.F. (2016). Osmoregulation and antioxidant production in maize under combined cadmium and arsenic stress. Environ. Sci. Pollut. Res..

[22] Taji T., Ohsumi C., Iuchi S., Seki M., Kasuga M., Kobayashi M., Yamaguchi-Shinozaki K., Shinozaki K. (2002). Important roles of drought-and cold-inducible genes for galactinol synthase in stress tolerance in *Arabidopsis thaliana*. Plant J..

[23] Khan M.I., Iqbal N., Masood A., Per T.S., Khan N.A. (2013). Salicylic acid alleviates adverse effects of heat stress on photosynthesis through changes in proline production and ethylene formation. Plant Signal. Behav..

[24] Shahzad B., Cheema S., Farooq M., Cheema Z., Rehman A., Abbas T. (2018). Growth Stimulating Influence of Foliage Applied *Brassica* Water Extracts on Morphological and Yield Attributes of Bread Wheat under Different Fertilizer Regimes. Planta Daninha.

[25] Fahad S., Hussain S., Saud S., Hassan S., Chauhan B.S., Khan F., Ihsan M.Z., Ullah A., Wu C., Bajwa A.A. (2016). Responses of Rapid Viscoanalyzer Profile and Other Rice Grain Qualities to Exogenously Applied Plant Growth Regulators under High Day and High Night Temperatures. PLoS ONE.

[26] Sharma A., Kumar V., Singh R., Thukral A.K., Bhardwaj R. (2016). Effect of seed pre-soaking with 24-epibrassinolide on growth and photosynthetic parameters of *Brassica juncea* L. in imidacloprid soil. Ecotoxicol. Environ. Saf..

[27] Sharma A., Zheng B. (2019). Melatonin Mediated Regulation of Drought Stress: Physiological and Molecular Aspects. Plants.

[28] Sharma A., Soares C., Sousa B., Martins M., Kumar V., Shahzad B., Sidhu G.P.S., Bali A.S., Asgher M., Bhardwaj R. (2019). Nitric oxide-mediated regulation of oxidative stress in plants under metal stress: A review on molecular and biochemical aspects. Physiol. Plant..

[29] Singh M., Kumar J., Singh S., Singh V.P., Prasad S.M. (2015). Roles of osmoprotectants in improving salinity and drought tolerance in plants: A review. Rev. Environ. Sci. Biotechnol..

[30] Wani S.H., Singh N.B., Haribhushan A., Mir J.I. (2013). Compatible solute engineering in plants for abiotic stress tolerance-role of glycine betaine. Curr. Genom..

[31] Alia P., Pardha S., Prasanna M. (1993). Proline in relation to free radical production in seedlings of *Brassica juncea* raised under sodium chloride stress. Plant Soil.

[32] Yancey P.H., Strange K. (1994). Compatible and counteracting solutes. Cellular and Molecular Physiology of Cell Volume Regulation.

[33] Ajithkumar I.P., Panneerselvam R. (2014). ROS Scavenging System, Osmotic Maintenance, Pigment and Growth Status of Panicum sumatrense Roth. Under Drought Stress. Cell Biochem. Biophys..

[34] Anjum N.A., Aref I.M., Duarte A.C., Pereira E., Ahmad I., Iqbal M. (2017). Glutathione and proline can coordinately make plants withstand the joint attack of metal (loid) and salinity stresses. Front. Plant Sci..

[35] Wang Y.-M., Meng Y.-L., Nii N. (2004). Changes in glycine betaine and related enzyme contents in Amaranthus tricolor under salt stress. J. Plant Physiol. Mol. Biol..

[36] Conde A., Silva P., Agasse A., Conde C., Gerós H. (2011). Mannitol transport and mannitol dehydrogenase activities are coordinated in olea europaea under salt and osmotic stresses. Plant Cell Physiol..

[37] Sharma S.S., Dietz K.-J. (2006). The significance of amino acids and amino acid-derived molecules in plant responses and adaptation to heavy metal stress. J. Exp. Bot..

[38] Hayashi H., Chen T.H.H., Murata N. (1998). Transformation with a gene for choline oxidase enhances the cold tolerance of *Arabidopsis* during germination and early growth. Plant Cell Environ..

[39] Ningthoujam M., Habib K., Bano F., Zutshi S., Fatma T. (2013). Exogenous osmolytes suppresses the toxic effects of malathion on *Anabaena variabilis*. Ecotoxicol. Environ. Saf..

[40] Jaleel C.A., Gopi R., Sankar B., Gomathinayagam M., Panneerselvam R. (2008). Differential responses in water use efficiency in two varieties of Catharanthus roseus under drought stress. Comptes Rendus Biol..

[41] Din J., Khan S., Ali I., Gurmani A. (2011). Physiological and agronomic response of canola varieties to drought stress. J. Anim. Plant Sci..

[42] Ashraf M., Karim F. (1991). Screening of some cultivars/lines of black gram (*Vigna mungo* L. Hepper) for resistance to water stress. Trop. Agric..

[43] Serraj R., Sinclair T. (2002). Osmolyte accumulation: Can it really help increase crop yield under drought conditions?. Plant Cell Environ..

[44] Alexieva V., Sergiev I., Mapelli S., Karanov E. (2001). The effect of drought and ultraviolet radiation on growth and stress markers in pea and wheat. Plant Cell Environ..

[45] Yamada M., Morishita H., Urano K., Shiozaki N., Yamaguchi-Shinozaki K., Shinozaki K., Yoshiba Y. (2005). Effects of free proline accumulation in petunias under drought stress. J. Exp. Bot..

[46] Quan R., Shang M., Zhang H., Zhao Y., Zhang J. (2004). Engineering of enhanced glycine betaine synthesis improves drought tolerance in maize. Plant Biotechnol. J..

[47] Naidu B.P. (1998). Simultaneous estimation of sugars, polyols, proline analogues and betaines accumulating in stressed plants by high performance liquid chromatography-Ultra violet detection. Aust. J. Plant Physiol..

[48] Akashi K., Miyake C., Yokota A. (2001). Citrulline, a novel compatible solute in drought-tolerant wild watermelon leaves, is an efficient hydroxyl radical scavenger. FEBS Lett..

[49] Chen X., Qiu L., Guo H., Wang Y., Yuan H., Yan D., Zheng B. (2017). Spermidine induces physiological and biochemical changes in southern highbush blueberry under drought stress. Braz. J. Bot..

[50] Cano E.A., Pérez-Alfocea F., Caro M., Bolarín M.C., Moreno V. (1998). Evaluation of salt tolerance in cultivated and wild tomato species through in vitro shoot apex culture. Plant Cell Tissue Organ Cult..

[51] Khan M.I., Asgher M., Khan N.A. (2014). Alleviation of salt-induced photosynthesis and growth inhibition by salicylic acid involves glycinebetaine and ethylene in mungbean (*Vigna radiata* L.). Plant Physiol. Biochem..

[52] Nazar R., Khan M.I., Iqbal N., Masood A., Khan N.A. (2014). Involvement of ethylene in reversal of salt-inhibited photosynthesis by sulfur in mustard. Physiol. Plant..

[53] Tomescu D., Şumălan R., Copolovici L., Copolovici D. (2017). The influence of soil salinity on volatile organic compounds emission and photosynthetic parameters of *Solanum lycopersicum* L. varieties. Open Life Sci..

[54] Tang W., Luo C. (2018). Overexpression of Zinc Finger Transcription Factor ZAT6 Enhances Salt Tolerance. Open Life Sci..

[55] Zhu J.-K. (2001). Plant salt tolerance. Trends Plant Sci..

[56] Sudhakar C., Lakshmi A., Giridarakumar S. (2001). Changes in the antioxidant enzyme efficacy in two high yielding genotypes of mulberry (*Morus alba* L.) under NaCl salinity. Plant Sci..

[57] Sumithra K., Jutur P., Carmel B.D., Reddy A.R. (2006). Salinity-induced changes in two cultivars of *Vigna radiata*: Responses of antioxidative and proline metabolism. Plant Growth Regul..

[58] Ahmad P., Jaleel C.A., Salem M.A., Nabi G., Sharma S. (2010). Roles of enzymatic and nonenzymatic antioxidants in plants during abiotic stress. Crit. Rev. Biotechnol..

[59] Hasanuzzaman M., Nahar K., Fujita M., Ahmad P., Azooz M.M., Prasad M.N.V. (2013). Plant Response to Salt Stress and Role of Exogenous Protectants to Mitigate Salt-Induced Damages. Ecophysiology and Responses of Plants under Salt Stress.

[60] Khedr A.H., Abbas M.A., Wahid A.A., Quick W.P., Abogadallah G.M. (2003). Proline induces the expression of salt-stress-responsive proteins and may improve the adaptation of *Pancratium maritimum* L. to salt-stress. J. Exp. Bot..

[61] Hoque M.A., Banu M.N., Nakamura Y., Shimoishi Y., Murata Y. (2008). Proline and glycinebetaine enhance antioxidant defense and methylglyoxal detoxification systems and reduce NaCl-induced damage in cultured tobacco cells. J. Plant Physiol..

[62] Chen T.H., Murata N. (2008). Glycinebetaine: An effective protectant against abiotic stress in plants. Trends Plant Sci..

[63] Mäkelä P., Kärkkäinen J., Somersalo S. (2000). Effect of Glycinebetaine on Chloroplast Ultrastructure, Chlorophyll and Protein Content, and RuBPCO Activities in Tomato Grown under Drought or Salinity. Biol. Plant..

[64] Ashraf M., Foolad M.R. (2007). Roles of glycine betaine and proline in improving plant abiotic stress resistance. Environ. Exp. Bot..

[65] Lutts S. (2000). Exogenous glycinebetaine reduces sodium accumulation in salt-stressed rice plants. Int. Rice Res. Notes.

[66] Reda F., Mandoura H.M.H. (2011). Response of enzymes activities, photosynthetic pigments, proline to low or high temperature stressed wheat plant (*Triticum aestivum* L.) in the presence or absence of exogenous proline or cysteine. Int. J. Acad. Res..

[67] Li J., Pandeya D., Nath K., Zulfugarov I.S., Yoo S.C., Zhang H., Yoo J.H., Cho S.H., Koh H.J., Kim D.S. (2010). ZEBRA-NECROSIS, a thylakoid-bound protein, is critical for the photoprotection of developing chloroplasts during early leaf development. Plant J..

[68] Tewari A.K., Tripathy B.C. (1998). Temperature-stress-induced impairment of chlorophyll biosynthetic reactions in cucumber and wheat. Plant Physiol..

[69] Xu Z.-S., Xia L.-Q., Chen M., Cheng X.-G., Zhang R.-Y., Li L.-C., Zhao Y.-X., Lu Y., Ni Z.-Y., Liu L. (2007). Isolation and molecular characterization of the *Triticum aestivum* L. ethylene-responsive factor 1 (TaERF1) that increases multiple stress tolerance. Plant Mol. Biol..

[70] Kishor P.K., Sangam S., Amrutha R., Laxmi P.S., Naidu K., Rao K., Rao S., Reddy K., Theriappan P., Sreenivasulu N. (2005). Regulation of proline biosynthesis, degradation, uptake and transport in higher plants: Its implications in plant growth and abiotic stress tolerance. Curr. Sci..

[71] Hayat S., Hayat Q., Alyemeni M.N., Wani A.S., Pichtel J., Ahmad A. (2012). Role of proline under changing environments: A review. Plant Signal. Behav..

[72] Vaida J., Natalija B., Ramune K., Ausra B. (2012). Effect of exogenous proline and de-acclimation treatment on cold tolerance in Brassica napus shoots cultured in vitro. J. Food Agric. Environ..

[73] Kawakami A., Sato Y., Yoshida M. (2008). Genetic engineering of rice capable of synthesizing fructans and enhancing chilling tolerance. J. Exp. Bot..

[74] Kathuria H., Giri J., Nataraja K.N., Murata N., Udayakumar M., Tyagi A.K. (2009). Glycinebetaine-induced water-stress tolerance in codA-expressing transgenic indica rice is associated with up-regulation of several stress responsive genes. Plant Biotechnol. J..

[75] Kohli S.K., Handa N., Sharma A., Gautam V., Arora S., Bhardwaj R., Alyemeni M.N., Wijaya L., Ahmad P. (2018). Combined effect of 24-epibrassinolide and salicylic acid mitigates lead (Pb) toxicity by modulating various metabolites in *Brassica juncea* L. seedlings. Protoplasma.

[76] Sharma J., Chakraverty N., Das A.B., Rout G.R. (2013). Mechanism of plant tolerance in response to heavy metals. Molecular Stress Physiology of Plants.

[77] Fahad S., Rehman A., Shahzad B., Tanveer M., Saud S., Kamran M., Ihtisham M., Khan S.U., Turan V., ur Rahman M.H., Hasanuzzaman M., Fujita M., Nahar K., Biswas J.K. (2019). Rice Responses and Tolerance to Metal/Metalloid Toxicity. Advances in Rice Research for Abiotic Stress Tolerance.

[78] Guo H., Chen H., Hong C., Jiang D., Zheng B. (2017). Exogenous malic acid alleviates cadmium toxicity in *Miscanthus sacchariflorus* through enhancing photosynthetic capacity and restraining ROS accumulation. Ecotoxicol. Environ. Saf..

[79] Nagajyoti P.C., Lee K.D., Sreekanth T.V.M. (2010). Heavy metals, occurrence and toxicity for plants: A review. Environ. Chem. Lett..

[80] Dugardeyn J., Van Der Straeten D. (2008). Ethylene: Fine-tuning plant growth and development by stimulation and inhibition of elongation. Plant Sci..

[81] Guo H., Feng X., Hong C., Chen H., Zeng F., Zheng B., Jiang D. (2017). Malate secretion from the root system is an important reason for higher resistance of *Miscanthus sacchariflorus* to cadmium. Physiol. Plant..

[82] Sidhu G.P.S., Singh H.P., Batish D.R., Kohli R.K. (2017). Appraising the role of environment friendly chelants in alleviating lead by *Coronopus didymus* from Pb-contaminated soils. Chemosphere.

[83] Malik A. (2004). Metal bioremediation through growing cells. Environ. Int..

[84] Dhir B., Nasim S.A., Samantary S., Srivastava S. (2012). Assessment of Osmolyte Accumulation in Heavy Metal Exposed Salvinia natans. Int. J. Bot..

[85] Bhatti K.H., Anwar S., Nawaz K., Hussain K., Siddiqi E., Sharif R., Talat A., Khalid A. (2013). Effect of exogenous application of glycinebetaine on wheat (*Triticum aestivum* L.) under heavy metal stress. Middle East J. Sci. Res..

[86] Kulheim C., Agren J., Jansson S. (2002). Rapid regulation of light harvesting and plant fitness in the field. Science.

[87] Kirchhoff H. (2014). Structural changes of the thylakoid membrane network induced by high light stress in plant chloroplasts. Philos. Trans. R. Soc. B Biol. Sci..

[88] Miyazawa S., Livingston N.J., Turpin D.H. (2006). Stomatal development in new leaves is related to the stomatal conductance of mature leaves in poplar (*Populus trichocarpa* x *P. deltoides*). J. Exp. Bot..

[89] Hideg E., Jansen M.A., Strid A. (2013). UV-B exposure, ROS, and stress: Inseparable companions or loosely linked associates?. Trends Plant Sci..

[90] Wang Y., Zhang H., Hou P., Su X., Zhao P., Zhao H., Liu S. (2014). Foliar-applied salicylic acid alleviates heat and high light stress induced photoinhibition in wheat (*Triticum aestivum*) during the grain filling stage by modulating the psbA gene transcription and antioxidant defense. Plant Growth Regul..

[91] Fujioka S., Yokota T. (2003). Biosynthesis and Metabolism of Brassinosteroids. Annu. Rev. Plant Biol..

[92] Vardhini B.V., Anuradha S., Sujatha E., Rao S.S.R. (2010). Role of brassinosteroids in alleviating various abiotic and biotic stresses-a review. Plant Nutr. Abiotic Stress Toler. I Plant Stress.

[93] Choudhary S.P., Oral H.V., Bhardwaj R., Yu J.-Q., Tran L.-S.P. (2012). Interaction of brassinosteroids and polyamines enhances copper stress tolerance in *Raphanus sativus*. J. Exp. Bot..

[94] Rattan A., Kapoor D., Kapoor N., Bhardwaj R. (2014). Application of brassionsteroids reverses the inhibitory effect of salt stress on growth and photosynthetic activity of *Zea mays* plants. Int. J. Theor. Appl. Sci..

[95] Coll Y., Coll F., Amoros A., Pujol M. (2015). Brassinosteroids roles and applications: An up-date. Biologia.

[96] Kohli S.K., Handa N., Bali S., Arora S., Sharma A., Kaur R., Bhardwaj R. (2018). Modulation of antioxidative defense expression and osmolyte content by co-application of 24-epibrassinolide and salicylic acid in Pb exposed Indian mustard plants. Ecotoxicol. Environ. Saf..

[97] Kohli S.K., Handa N., Sharma A., Gautam V., Arora S., Bhardwaj R., Wijaya L., Alyemeni M.N., Ahmad P. (2018). Interaction of 24-epibrassinolide and salicylic acid regulates pigment contents, antioxidative defense responses, and gene expression in *Brassica juncea* L. seedlings under Pb stress. Environ. Sci. Pollut. Res..

[98] Sharma A., Kumar V., Kumar R., Shahzad B., Thukral A.K., Bhardwaj R. (2018). Brassinosteroid-mediated pesticide detoxification in plants: A mini-review. Cogent Food Agric..

[99] Rehman S., Shahzad B., Bajwa A.A., Hussain S., Rehman A., Cheema S.A., Abbas T., Ali A., Shah L., Adkins S. (2019). Utilizing the Allelopathic Potential of *Brassica* Species for Sustainable Crop Production: A Review. J. Plant Growth Regul..

[100] Sharma A., Kumar V., Kanwar M.K., Thukral A.K., Bhardwaj R. (2017). Ameliorating imidacloprid induced oxidative stress by 24-epibrassinolide in *Brassica juncea* L.. Russ. J. Plant Physiol..

[101] Handa N., Kohli S.K., Kaur R., Sharma A., Kumar V., Thukral A.K., Arora S., Bhardwaj R., Hasanuzzaman M., Nahar K., Fujita M. (2018). Role of Compatible Solutes in Enhancing Antioxidative Defense in Plants Exposed to Metal Toxicity. Plants under Metal and Metalloid Stress: Responses, Tolerance and Remediation.

[102] Kaur R., Yadav P., Sharma A., Kumar Thukral A., Kumar V., Kaur Kohli S., Bhardwaj R. (2017). Castasterone and citric acid treatment restores photosynthetic attributes in *Brassica juncea* L. under Cd(II) toxicity. Ecotoxicol. Environ. Saf..

[103] Fàbregas N., Lozano-Elena F., Blasco-Escámez D., Tohge T., Martínez-Andújar C., Albacete A., Osorio S., Bustamante M., Riechmann J.L., Nomura T. (2018). Overexpression of the vascular brassinosteroid receptor BRL3 confers drought resistance without penalizing plant growth. Nat. Commun..

[104] Verbruggen N., Hermans C. (2008). Proline accumulation in plants: A review. Amino Acids.

[105] Kido E.A., Neto J.R.F., Silva R.L., Belarmino L.C., Neto J.P.B., Soares-Cavalcanti N.M., Pandolfi V., Silva M.D., Nepomuceno A.L., Benko-Iseppon A.M. (2013). Expression dynamics and genome distribution of osmoprotectants in soybean: Identifying important components to face abiotic stress. BMC Bioinform..

[106] Kumar K., Kumar M., Kim S.-R., Ryu H., Cho Y.-G. (2013). Insights into genomics of salt stress response in rice. Rice.

[107] Choudhary S.P., Bhardwaj R., Gupta B.D., Dutt P., Gupta R.K., Biondi S., Kanwar M. (2010). Epibrassinolide induces changes in indole-3-acetic acid, abscisic acid and polyamine concentrations and enhances antioxidant potential of radish seedlings under copper stress. Physiol. Plant..

[108] Sharma I., Pati P.K., Bhardwaj R. (2011). Effect of 24-epibrassinolide on oxidative stress markers induced by nickel-ion in *Raphanus sativus* L.. Acta Physiol. Plant..

[109] Fariduddin Q., Khalil R.R., Mir B.A., Yusuf M., Ahmad A. (2013). 24-Epibrassinolide regulates photosynthesis, antioxidant enzyme activities and proline content of *Cucumis sativus* under salt and/or copper stress. Environ. Monit. Assess..

[110] Zhu J., Lu P., Jiang Y., Wang M., Zhang L. (2014). Effects of brassinosteroid on antioxidant system in *Salvia miltiorrhiza* under drought stress. J. Res. Agric. Anim. Sci..

[111] Khamsuk O., Sonjaroon W., Suwanwong S., Jutamanee K., Suksamrarn A. (2018). Effects of 24-epibrassinolide and the synthetic brassinosteroid mimic on chili pepper under drought. Acta Physiol. Plant..

[112] Fariduddin Q., Yusuf M., Hayat S., Ahmad A. (2009). Effect of 28-homobrassinolide on antioxidant capacity and photosynthesis in Brassica juncea plants exposed to different levels of copper. Environ. Exp. Bot..

[113] Lalotra S., Hemantaranjan A., Kumar S., Kant R. (2017). Effect of brassinosteroid (brassinolide) on seedling traits, morphology and metabolism in mung bean under salinity stress. Annu. Res. Rev. Biol..

[114] Gao H., Zhang Z., Lv X., Cheng N., Peng B., Cao W. (2016). Effect of 24-epibrassinolide on chilling injury of peach fruit in relation to phenolic and proline metabolisms. Postharvest Biol. Technol..

[115] Hare P., Cress W., Van Staden J. (1999). Proline synthesis and degradation: A model system for elucidating stress-related signal transduction. J. Exp. Bot..

[116] Delauney A.J., Verma D.P.S. (1993). Proline biosynthesis and osmoregulation in plants. Plant J..

[117] Wani A.S., Ahmad A., Hayat S., Tahir I. (2019). Epibrassinolide and proline alleviate the photosynthetic and yield inhibition under salt stress by acting on antioxidant system in mustard. Plant Physiol. Biochem..

[118] Kurepin L.V., Ozga J.A., Zaman M., Pharis R.P. (2013). The physiology of plant hormones in cereal, oilseed and pulse crops. Prairie Soils Crop..

[119] Kurepin L.V., Ivanov A.G., Zaman M., Pharis R.P., Allakhverdiev S.I., Hurry V., Huner N.P. (2015). Stress-related hormones and glycinebetaine interplay in protection of photosynthesis under abiotic stress conditions. Photosynth. Res..

[120] Huang J., Hirji R., Adam L., Rozwadowski K.L., Hammerlindl J.K., Keller W.A., Selvaraj G. (2000). Genetic engineering of glycinebetaine production toward enhancing stress tolerance in plants: metabolic limitations. Plant Physiol..

[121] Kurepin L.V., Ivanov A.G., Zaman M., Pharis R.P., Hurry V., Hüner N.P., Hou H.J.M., Najafpour M.M., Moore G.F., Allakhverdiev S.I. (2017). Interaction of glycine betaine and plant hormones: Protection of the photosynthetic apparatus during abiotic stress. Photosynthesis: Structures, Mechanisms, and Applications.

[122] Park E.J., Jeknić Z., Pino M.T., Murata N., Chen T.H.H. (2007). Glycinebetaine accumulation is more effective in chloroplasts than in the cytosol for protecting transgenic tomato plants against abiotic stress. Plant Cell Environ..

[123] Mäkelä P., Peltonen-Sainio P., Jokinen K., Pehu E., Setälä H., Hinkkanen R., Somersalo S. (1996). Uptake and translocation of foliar-applied glycinebetaine in crop plants. Plant Sci..

[124] Hattori T., Mitsuya S., Fujiwara T., Jagendorf A.T., Takabe T. (2009). Tissue specificity of glycinebetaine synthesis in barley. Plant Sci..

[125] Adams W.W., Muller O., Cohu C.M., Demmig-Adams B. (2013). May photoinhibition be a consequence, rather than a cause, of limited plant productivity?. Photosynth. Res..

[126] Choudhary S.P., Kanwar M., Bhardwaj R., Gupta B., Gupta R. (2011). Epibrassinolide ameliorates Cr (VI) stress via influencing the levels of indole-3-acetic acid, abscisic acid, polyamines and antioxidant system of radish seedlings. Chemosphere.

[127] Shahid M.A., Balal R.M., Pervez M.A., Garcia-Sanchez F., Gimeno V., Abbas T., Mattson N.S., Riaz A. (2014). Treatment with 24-epibrassinolide mitigates NaCl-induced toxicity by enhancing carbohydrate metabolism, osmolyte accumulation, and antioxidant activity in *Pisum sativum*. Turk. J. Bot..

[128] Ahmad P., Abd_Allah E.F., Alyemeni M.N., Wijaya L., Alam P., Bhardwaj R., Siddique K.H. (2018). Exogenous application of calcium to 24-epibrassinosteroid pre-treated tomato seedlings mitigates NaCl toxicity by modifying ascorbate–glutathione cycle and secondary metabolites. Sci. Rep..

[129] Jan S., Alyemeni M.N., Wijaya L., Alam P., Siddique K.H., Ahmad P. (2018). Interactive effect of 24-epibrassinolide and silicon alleviates cadmium stress via the modulation of antioxidant defense and glyoxalase systems and macronutrient content in Pisum sativum L. seedlings. BMC Plant Biol..

[130] Qayyum B., Shahbaz M., Akram N.A. (2007). Interactive effect of foliar application of 24-epibrassinolide and root zone salinity on morpho-physiological attributes of wheat (*Triticum aestivum* L.). Int. J. Agric. Biol..

[131] Sengupta A., Chakraborty M., Saha J., Gupta B., Gupta K., Iqbal N., Nazar R., Khan N.A. (2016). Polyamines: Osmoprotectants in plant abiotic stress adaptation. Osmolytes and Plants Acclimation to Changing Environment: Emerging Omics Technologies.

[132] Liu J.-H., Wang W., Wu H., Gong X., Moriguchi T. (2015). Polyamines function in stress tolerance: From synthesis to regulation. Front. Plant Sci..

[133] Yang J., Zhang J., Liu K., Wang Z., Liu L. (2007). Involvement of polyamines in the drought resistance of rice. J. Exp. Bot..

[134] Takahashi T., Kakehi J.-I. (2009). Polyamines: Ubiquitous polycations with unique roles in growth and stress responses. Ann. Bot..

[135] Kuznetsov V.V., Shevyakova N.I., Ramawat K.G. (2010). Polyamines and Plant Adaptation to Saline Environments. Desert Plants: Biology and Biotechnology.

[136] Parvaiz A., Satyawati S. (2008). Salt stress and phyto-biochemical responses of plants-a review. Plant Soil Environ..

[137] Murakeozy E.P., Nagy Z., Duhaze C., Bouchereau A., Tuba Z. (2003). Seasonal changes in the levels of compatible osmolytes in three halophytic species of inland saline vegetation in Hungary. J. Plant Physiol..

[138] Zhang Y., He J. (2015). Sugar-induced plant growth is dependent on brassinosteroids. Plant Signal. Behav..

[139] Zhang Y., Liu Z., Wang J., Chen Y., Bi Y., He J. (2015). Brassinosteroid is required for sugar promotion of hypocotyl elongation in *Arabidopsis* in darkness. Planta.

[140] Osborne D.J. (1993). Ethylene in Plant Biology.

[141] Cao W.-H., Liu J., He X.-J., Mu R.-L., Zhou H.-L., Chen S.-Y., Zhang J.-S. (2007). Modulation of ethylene responses affects plant salt-stress responses. Plant Physiol..

[142] Chen Y.-F., Etheridge N., Schaller G.E. (2005). Ethylene signal transduction. Ann. Bot..

[143] Chen T., Zhang J.S. (2006). Ethylene biosynthesis and signaling pathway. Chin. Bull. Bot..

[144] Hattori Y., Nagai K., Furukawa S., Song X.-J., Kawano R., Sakakibara H., Wu J., Matsumoto T., Yoshimura A., Kitano H. (2009). The ethylene response factors SNORKEL1 and SNORKEL2 allow rice to adapt to deep water. Nature.

[145] Iqbal N., Umar S., Khan N.A. (2015). Nitrogen availability regulates proline and ethylene production and alleviates salinity stress in mustard (*Brassica juncea*). J. Plant Physiol..

[146] Cui M., Lin Y., Zu Y., Efferth T., Li D., Tang Z. (2015). Ethylene increases accumulation of compatible solutes and decreases oxidative stress to improve plant tolerance to water stress in *Arabidopsis*. J. Plant Biol..

[147] Filippou P., Bouchagier P., Skotti E., Fotopoulos V. (2014). Proline and reactive oxygen/nitrogen species metabolism is involved in the tolerant response of the invasive plant species *Ailanthus altissima* to drought and salinity. Environ. Exp. Bot..

[148] Nounjan N., Nghia P.T., Theerakulpisut P. (2012). Exogenous proline and trehalose promote recovery of rice seedlings from salt-stress and differentially modulate antioxidant enzymes and expression of related genes. J. Plant Physiol..

[149] Shahbaz M., Mushtaq Z., Andaz F., Masood A. (2013). Does proline application ameliorate adverse effects of salt stress on growth, ions and photosynthetic ability of eggplant (*Solanum melongena* L.)?. Sci. Hortic..

[150] Iqbal N., Umar S., Khan N.A., Khan M.I.R. (2014). A new perspective of phytohormones in salinity tolerance: Regulation of proline metabolism. Environ. Exp. Bot..

[151] Khan M.I., Nazir F., Asgher M., Per T.S., Khan N.A. (2015). Selenium and sulfur influence ethylene formation and alleviate cadmium-induced oxidative stress by improving proline and glutathione production in wheat. J. Plant Physiol..

[152] Chrominski A., Halls S., Weber D., Smith B. (1989). Proline affects ACC to ethylene conversion under salt and water stresses in the halophyte, *Allenrolfea occidentalis*. Environ. Exp. Bot..

[153] Ranganayakulu G.S., Veeranagamallaiah G., Sudhakar C. (2013). Effect of salt stress on osmolyte accumulation in two groundnut cultivars (*Arachis hypogaea* L.) with contrasting salt tolerance. Afr. J. Plant Sci..

[154] Walton L.J., Kurepin L.V., Yeung E.C., Shah S., Emery R.N., Reid D.M., Pharis R.P. (2012). Ethylene involvement in silique and seed development of canola, *Brassica napus* L.. Plant Physiol. Biochem..

[155] Morgan P.W., Drew M.C. (1997). Ethylene and plant responses to stress. Physiol. Plant..

[156] Park E.J., Jeknic Z., Sakamoto A., DeNoma J., Yuwansiri R., Murata N., Chen T.H. (2004). Genetic engineering of glycinebetaine synthesis in tomato protects seeds, plants, and flowers from chilling damage. Plant J..

[157] Wang G., Li F., Zhang J., Zhao M., Hui Z., Wang W. (2010). Overaccumulation of glycine betaine enhances tolerance of the photosynthetic apparatus to drought and heat stress in wheat. Photosynthetica.

[158] Allard F., Houde M., Sarhan F., Kröl M., Ivanov A., Huner N.P.A. (1998). Betaine Improves Freezing Tolerance in Wheat. Plant Cell Physiol..

[159] Quan R., Shang M., Zhang H., Zhao Y., Zhang J. (2004). Improved chilling tolerance by transformation with betA gene for the enhancement of glycinebetaine synthesis in maize. Plant Sci..

[160] Hussain S.S., Ali M., Ahmad M., Siddique K.H. (2011). Polyamines: Natural and engineered abiotic and biotic stress tolerance in plants. Biotechnol. Adv..

[161] Kubiś J., Floryszak-Wieczorek J., Arasimowicz-Jelonek M. (2014). Polyamines induce adaptive responses in water deficit stressed cucumber roots. J. Plant Res..

[162] Petruzzelli L., Coraggio I., Leubner-Metzger G. (2000). Ethylene promotes ethylene biosynthesis during pea seed germination by positive feedback regulation of 1-aminocyclo-propane-1-carboxylic acid oxidase. Planta.

[163] Li C.-Z., Jiao J., Wang G.-X. (2004). The important roles of reactive oxygen species in the relationship between ethylene and polyamines in leaves of spring wheat seedlings under root osmotic stress. Plant Sci..

[164] Li C.-z., Wang G.-x. (2004). Interactions between reactive oxygen species, ethylene and polyamines in leaves of *Glycyrrhiza inflata* seedlings under root osmotic stress. Plant Growth Regul..

[165] Van den Ende W., Valluru R. (2008). Sucrose, sucrosyl oligosaccharides, and oxidative stress: Scavenging and salvaging?. J. Exp. Bot..

[166] Koyro H.-W., Ahmad P., Geissler N., Ahmad P., Prasad M.N.V. (2012). Abiotic Stress Responses in Plants: An Overview. Environmental Adaptations and Stress Tolerance of Plants in the Era of Climate Change.

[167] Kerepesi I., Galiba G. (2000). Osmotic and Salt Stress-Induced Alteration in Soluble Carbohydrate Content in Wheat Seedlings. Crop Sci..

[168] Pilon-Smits E.A.H., Terry N., Sears T., van Dun K. (1999). Enhanced drought resistance in fructan-producing sugar beet. Plant Physiol. Biochem..

[169] Luo Y., Li F., Wang G., Yang X., Wang W. (2010). Exogenously-supplied trehalose protects thylakoid membranes of winter wheat from heat-induced damage. Biol. Plant..

[170] Theerakulpisut P., Gunnula W. (2012). Exogenous sorbitol and trehalose mitigated salt stress damage in salt-sensitive but not salt-tolerant rice seedlings. Asian J. Crop Sci..

[171] Li H.W., Zang B.S., Deng X.W., Wang X.P. (2011). Overexpression of the trehalose-6-phosphate synthase gene OsTPS1 enhances abiotic stress tolerance in rice. Planta.

[172] Kanayama Y., Watanabe M., Moriguchi R., Deguchi M., Kanahama K., Yamaki S. (2006). Effects of Low Temperature and Abscisic Acid on the Expression of the Sorbitol-6-phosphate Dehydrogenase Gene in Apple Leaves. J. Jpn. Soc. Hortic. Sci..

[173] Patonnier M.P., Peltier J.P., Marigo G. (1999). Drought-induced increase in xylem malate and mannitol concentrations and closure of *Fraxinus excelsior* L. stomata. J. Exp. Bot..

[174] Kaya C., Sonmez O., Aydemir S., Ashraf M., Dikilitas M. (2013). Exogenous application of mannitol and thiourea regulates plant growth and oxidative stress responses in salt-stressed maize (*Zea mays* L.). J. Plant Interact..

[175] Ahn C., Park U., Park P.B. (2011). Increased salt and drought tolerance by d-ononitol production in transgenic *Arabidopsis thaliana*. Biochem. Biophy. Res. Commun..

[176] Misra N., Saxena P. (2009). Effect of salicylic acid on proline metabolism in lentil grown under salinity stress. Plant Sci..

[177] Misra N., Misra R. (2012). Salicylic acid changes plant growth parameters and proline metabolism in *Rauwolfia serpentina* leaves grown under salinity stress. Am. Eurasian J. Agric. Environ. Sci..

[178] Zengin F. (2014). Exogenous treatment with salicylic acid alleviating copper toxicity in bean seedlings. Proceed. Nat. Acad. Sci. Ind. Sec. B Biol. Sci..

[179] Yuan Z., Cong G., Zhang J. (2014). Effects of exogenous salicylic acid on polysaccharides production of *Dendrobium officinale*. S. Afr. J. Bot..

[180] Chen T.H., Murata N. (2011). Glycinebetaine protects plants against abiotic stress: Mechanisms and biotechnological applications. Plant Cell Environ..

[181] Li T., Hu Y., Du X., Tang H., Shen C., Wu J. (2014). Salicylic acid alleviates the adverse effects of salt stress in *Torreya grandis* cv. Merrillii seedlings by activating photosynthesis and enhancing antioxidant systems. PLoS ONE.

[182] Hayat S., Masood A., Yusuf M., Fariduddin Q., Ahmad A. (2009). Growth of Indian mustard (*Brassica juncea* L.) in response to salicylic acid under high-temperature stress. Braz. J. Plant Physiol..

[183] Ma X., Zheng J., Zhang X., Hu Q., Qian R. (2017). Salicylic acid alleviates the adverse effects of salt stress on *Dianthus superbus* (Caryophyllaceae) by activating photosynthesis, protecting morphological structure, and enhancing the antioxidant system. Front. Plant Sci..

[184] La V.H., Lee B.-R., Zhang Q., Park S.-H., Islam M.T., Kim T.-H. (2019). Salicylic acid improves drought-stress tolerance by regulating the redox status and proline metabolism in *Brassica rapa*. Hortic. Environ. Biotechnol..

[185] Jagendorf A.T., Takabe T. (2001). Inducers of glycinebetaine synthesis in barley. Plant Physiol..

[186] Hoyos M.E., Zhang S. (2000). Calcium-independent activation of salicylic acid-induced protein kinase and a 40-kilodalton protein kinase by hyperosmotic stress. Plant Physiol..

[187] Aldesuquy H.S., Abbas M.A., Abo-Hamed S.A., Elhakem A.H., Alsokari S.S. (2012). Glycine betaine and salicylic acid induced modification in productivity of two different cultivars of wheat grown under water stress. J. Stress Physiol. Biochem..

[188] Hussain M., Malik M., Farooq M., Khan M., Akram M., Saleem M. (2009). Exogenous glycinebetaine and salicylic acid application improves water relations, allometry and quality of hybrid sunflower under water deficit conditions. J. Agron. Crop Sci..

[189] Gharbi E., Martínez J.-P., Benahmed H., Fauconnier M.-L., Lutts S., Quinet M. (2016). Salicylic acid differently impacts ethylene and polyamine synthesis in the glycophyte *Solanum lycopersicum* and the wild-related halophyte Solanum chilense exposed to mild salt stress. Physiol. Plant..

[190] Liu G., Ji Y., Bhuiyan N.H., Pilot G., Selvaraj G., Zou J., Wei Y. (2010). Amino acid homeostasis modulates salicylic acid–associated redox status and defense responses in *Arabidopsis*. Plant Cell.

[191] van Damme M., Zeilmaker T., Elberse J., Andel A., de Sain-van der Velden M., van den Ackerveken G. (2009). Downy mildew resistance in Arabidopsis by mutation of HOMOSERINE KINASE. Plant Cell.

[192] Farhangi-Abriz S., Ghassemi-Golezani K. (2016). Improving amino acid composition of soybean under salt stress by salicylic acid and jasmonic acid. J. Appl. Bot. Food Qual..

[193] Zushi K. (2005). Comparison of chemical composition contents of tomato fruit grown under water and salinity stresses. J. SHITA.

[194] Yadav S., Lakshmi N.J., Maheswari M., Vanaja M., Venkateswarlu B. (2005). Influence of water deficit at vegetative, anthesis and grain filling stages on water relation and grain yield in sorghum. Indian J. Plant Physiol..

[195] Sankar B., Jaleel C.A., Manivannan P., Kishorekumar A., Somasundaram R., Panneerselvam R. (2007). Drought-induced biochemical modifications and proline metabolism in *Abelmoschus esculentus* (L.) Moench. Acta Bot. Croat..

[196] El-Tayeb M., El-Enany A., Ahmed N. (2006). Salicylic acid-induced adaptive response to copper stress in sunflower (*Helianthus annuus* L.). Plant Growth Regul..

[197] Hussein M., Balbaa L., Gaballah M. (2007). Salicylic acid and salinity effects on growth of maize plants. Res. J. Agric. Biol. Sci..

[198] Abd Allah M.M.S., El-Bassiouny H.M.S., Elewa T.A.E., El-Sebai T.N. (2015). Effect of salicylic acid and benzoic acid on growth, yield and some biochemical aspects of quinoa plant grown in sandy soil. Int. J. Chem. Tech. Res..

[199] Khodary S. (2004). Effect of salicylic acid on the growth, photosynthesis and carbohydrate metabolism in salt stressed maize plants. Int. J. Agric. Biol..

[200] Balibrea M.E., Dell’Amico J., Bolaŕn M.C., Pérez-Alfocea F. (2000). Carbon partitioning and sucrose metabolism in tomato plants growing under salinity. Physiol. Plant..

[201] Bohnert H.J., Jensen R.G. (1996). Strategies for engineering water-stress tolerance in plants. Trends Biotechnol..

[202] Bakry B.A., El-Hariri D.M., Sadak M.S., El-Bassiouny H.M.S. (2012). Drought stress mitigation by foliar application of salicylic acid in two linseed varieties grown under newly reclaimed sandy soil. J. Appl. Sci. Res..

[203] Nazar R., Umar S., Khan N., Sareer O. (2015). Salicylic acid supplementation improves photosynthesis and growth in mustard through changes in proline accumulation and ethylene formation under drought stress. S. Afr. J. Bot..

[204] Nazar R., Umar S., Khan N.A. (2015). Exogenous salicylic acid improves photosynthesis and growth through increase in ascorbate-glutathione metabolism and S assimilation in mustard under salt stress. Plant Signal. Behav..

[205] Zhang Y., Xu S., Yang S., Chen Y. (2015). Salicylic acid alleviates cadmium-induced inhibition of growth and photosynthesis through upregulating antioxidant defense system in two melon cultivars (*Cucumis melo* L.). Protoplasma.

[206] Kotapati K.V., Palaka B.K., Ampasala D.R. (2017). Alleviation of nickel toxicity in finger millet (*Eleusine coracana* L.) germinating seedlings by exogenous application of salicylic acid and nitric oxide. Crop J..

[207] Chandrakar V., Dubey A., Keshavkant S. (2016). Modulation of antioxidant enzymes by salicylic acid in arsenic exposed *Glycine max* L.. J. Soil Sci. Plant Nutr..

[208] Ghasemi M., Modarresi M., Babaeian Jelodar N., Bagheri N., Jamali A. (2016). The evaluation of exogenous application of salicylic acid on physiological characteristics, proline and essential oil content of chamomile (*Matricaria chamomilla* L.) under normal and heat stress conditions. Agriculture.

[209] Zhou Z.S., Guo K., Elbaz A.A., Yang Z.M. (2009). Salicylic acid alleviates mercury toxicity by preventing oxidative stress in roots of *Medicago sativa*. Environ. Exp. Bot..

[210] Gaballah M., Rady M. (2012). Salicylic acid mitigated cadmium toxicity by attenuating the oxidative stress in pea (*Pisum sativum* L.) plants. Int. J. Biol. Ecol. Environ. Sci..

[211] Manaa A., Gharbi E., Mimouni H., Wasti S., Aschi-Smiti S., Lutts S., Ahmed H.B. (2014). Simultaneous application of salicylic acid and calcium improves salt tolerance in two contrasting tomato (*Solanum lycopersicum*) cultivars. S. Afr. J. Bot..

[212] Munir M., Shabbir G. (2018). Salicylic acid mediated heat stress tolerance in selected bread wheat genotypes of Pakistan. Pak. J. Bot..

[213] Kareem F., Rihan H., Fuller M. (2017). The effect of exogenous applications of salicylic acid and molybdenum on the tolerance of drought in wheat. Agric. Res. Technol..

[214] Zengin F. (2015). Effects of exogenous salicylic acid on growth characteristics and biochemical content of wheat seeds under arsenic stress. J. Environ. Biol..

[215] Al-Hakimi A.B.M., Hama A.M. (2011). Ascorbic acid, thiamine or salicylic acid induced changes in some physiological parameters in wheat grown under copper stress. Plant Prot. Sci..

[216] Li Z.-G. (2015). Synergistic effect of antioxidant system and osmolyte in hydrogen sulfide and salicylic acid crosstalk-induced heat tolerance in maize (*Zea mays* L.) seedlings. Plant Signal. Behav..

[217] Mohsenzadeh S., Shahrtash M., Mohabatkar H. (2011). Interactive effects of salicylic acid and silicon on some physiological responses of cadmium-stressed maize seedlings. Iran. J. Sci. Technol..

[218] Krantev A., Yordanova R., Janda T., Szalai G., Popova L. (2008). Treatment with salicylic acid decreases the effect of cadmium on photosynthesis in maize plants. J. Plant Physiol..

[219] Pavlů J., Novák J., Koukalová V., Luklová M., Brzobohatý B., Černý M. (2018). Cytokinin at the Crossroads of Abiotic Stress Signalling Pathways. Int. J. Mol. Sci..

[220] Kieber J.J., Schaller G.E. (2010). The perception of cytokinin: A story 50 years in the making. Plant Physiol..

[221] Schaller G.E., Street I.H., Kieber J.J. (2014). Cytokinin and the cell cycle. Curr. Opin. Plant Biol..

[222] Su Y.-H., Liu Y.-B., Zhang X.-S. (2011). Auxin–cytokinin interaction regulates meristem development. Mol. Plant.

[223] Zhang W., Swarup R., Bennett M., Schaller G.E., Kieber J.J. (2013). Cytokinin induces cell division in the quiescent center of the *Arabidopsis* root apical meristem. Curr. Biol..

[224] Bielach A., Podlešáková K., Marhavý P., Duclercq J., Cuesta C., Müller B., Grunewald W., Tarkowski P., Benková E. (2012). Spatiotemporal regulation of lateral root organogenesis in *Arabidopsis* by cytokinin. Plant Cell.

[225] Bishopp A., Help H., El-Showk S., Weijers D., Scheres B., Friml J., Benková E., Mähönen A.P., Helariutta Y. (2011). A mutually inhibitory interaction between auxin and cytokinin specifies vascular pattern in roots. Curr. Biol..

[226] Efroni I., Han S.-K., Kim H.J., Wu M.-F., Steiner E., Birnbaum K.D., Hong J.C., Eshed Y., Wagner D. (2013). Regulation of leaf maturation by chromatin-mediated modulation of cytokinin responses. Dev. Cell.

[227] Durán-Medina Y., Díaz-Ramírez D., Marsch-Martínez N. (2017). Cytokinins on the Move. Front. Plant Sci..

[228] Zwack P.J., Rashotte A.M. (2013). Cytokinin inhibition of leaf senescence. Plant Signal. Behav..

[229] Albacete A., Ghanem M.E., Martínez-Andújar C., Acosta M., Sánchez-Bravo J., Martínez V., Lutts S., Dodd I.C., Pérez-Alfocea F. (2008). Hormonal changes in relation to biomass partitioning and shoot growth impairment in salinized tomato (*Solanum lycopersicum* L.) plants. J. Exp. Bot..

[230] Alvarez S., Marsh E.L., Schroeder S.G., Schachtman D.P. (2008). Metabolomic and proteomic changes in the xylem sap of maize under drought. Plant Cell Environ..

[231] Shen X., Wang Z., Song X., Xu J., Jiang C., Zhao Y., Ma C., Zhang H. (2014). Transcriptomic profiling revealed an important role of cell wall remodeling and ethylene signaling pathway during salt acclimation in *Arabidopsis*. Plant Mol. Biol..

[232] Szabados L., Savoure A. (2010). Proline: A multifunctional amino acid. Trends Plant Sci..

[233] Rajendrakumar C.S., Reddy B.V., Reddy A.R. (1994). Proline-protein interactions: Protection of structural and functional integrity of M4 lactate dehydrogenase. Biochem. Biophy. Res. Commun..

[234] Mishra S., Dubey R.S. (2006). Inhibition of ribonuclease and protease activities in arsenic exposed rice seedlings: Role of proline as enzyme protectant. J. Plant Physiol..

[235] Thomas J.C., McElwain E.F., Bohnert H.J. (1992). Convergent induction of osmotic stress-responses: Abscisic acid, cytokinin, and the effects of NaCl. Plant Physiol..

[236] Türkan I., Demiral T. (2009). Recent developments in understanding salinity tolerance. Environ. Exp. Bot..

[237] Lokhande V.H., Suprasanna P., Ahmad P., Prasad M.N.V. (2012). Prospects of halophytes in understanding and managing abiotic stress tolerance. Environmental Adaptations and Stress Tolerance of Plants in the Era of Climate Change.

[238] Fitzgerald T.L., Waters D.L., Henry R.J. (2009). Betaine aldehyde dehydrogenase in plants. Plant Biol..

[239] Subbarao G.V., Wheeler R.M., Levine L.H., Stutte G.W. (2001). Glycine betaine accumulation, ionic and water relations of red-beet at contrasting levels of sodium supply. J. Plant Physiol..

[240] Huner N.P., Öquist G., Sarhan F. (1998). Energy balance and acclimation to light and cold. Trends Plant Sci..

[241] Rathinasabapathi B., Fouad W.M., Sigua C.A. (2001). beta-Alanine betaine synthesis in the Plumbaginaceae. Purification and characterization of a trifunctional, S-adenosyl-L-methionine-dependent N-methyltransferase from *Limonium latifolium* leaves. Plant Physiol..

[242] Hansen H., Dörffling K. (2003). Root-derived trans-zeatin riboside and abscisic acid in drought-stressed and rewatered sunflower plants: Interaction in the control of leaf diffusive resistance?. Funct. Plant Biol..

[243] Davies W.J., Kudoyarova G., Hartung W. (2005). Long-distance ABA signaling and its relation to other signaling pathways in the detection of soil drying and the mediation of the plant’s response to drought. J. Plant Growth Regul..

[244] Galston A.W. (1983). Polyamines as modulators of plant development. Bioscience.

[245] Wimalasekera R., Scherer G.F., Hayat S., Mori M., Pichtel J., Ahmad A. (2009). Polyamines and cytokinin: Is nitric oxide biosynthesis the key to overlapping functions?. Nitric Oxide in Plant Physiology.

[246] Suresh M.R., Ramakrishna S., Adiga P.R. (1978). Regulation of arginine decarboxylase and putrescine levels in *Cucumis sativus* cotyledons. Phytochemistry.

[247] Choudhuri M.M., Ghosh B. (1982). Purification and partial characterization of arginine decarboxylase from rice embryos (*Oryza sativa* L.). Agric. Biol. Chem..

[248] Palavan N., Goren R., Galston A.W. (1984). Effects of some growth regulators on polyamine biosynthetic enzymes in etiolated pea seedlings. Plant Cell Physiol..

[249] Legocka J., Żarnowska A. (2000). Role of polyamines in the cytokinin-dependent physiological processes II. Modulation of polyamine levels during cytokinin-stimulated expansion of cucumber cotyledons. Acta Physiol. Plant..

[250] Sobieszczuk-Nowicka E., Rorat T., Legocka J. (2007). Polyamine metabolism and S-adenosylmethionine decarboxylase gene expression during the cytokinin-stimulated greening process. Acta Physiol. Plant..

[251] Yuanyuan M., Yali Z., Jiang L., Hongbo S. (2009). Roles of plant soluble sugars and their responses to plant cold stress. Afr. J. Biotechnol..

[252] Hare P.D., Cress W.A., Van Staden J. (1998). Dissecting the roles of osmolyte accumulation during stress. Plant Cell Environ..

[253] Noiraud N., Maurousset L., Lemoine R. (2001). Transport of polyols in higher plants. Plant Physiol. Biochem..

[254] Lunn J.E., Delorge I., Figueroa C.M., Van Dijck P., Stitt M. (2014). Trehalose metabolism in plants. Plant J..

[255] Crowe J.H. (2007). Trehalose as a “chemical chaperone”: Fact and fantasy. Adv. Exp. Med. Biol..

[256] Loescher W.H., Tyson R.H., Everard J.D., Redgwell R.J., Bieleski R.L. (1992). Mannitol Synthesis in Higher Plants: Evidence for the Role and Characterization of a NADPH-Dependent Mannose 6-Phosphate Reductase. Plant Physiol..

[257] Llanes A., Bertazza G., Palacio G., Luna V. (2013). Different sodium salts cause different solute accumulation in the halophyte *Prosopis strombulifera*. Plant Biol..

[258] Bali S., Kaur P., Sharma A., Ohri P., Bhardwaj R., Alyemeni M.N., Wijaya L., Ahmad P. (2018). Jasmonic acid-induced tolerance to root-knot nematodes in tomato plants through altered photosynthetic and antioxidative defense mechanisms. Protoplasma.

[259] Per T.S., Khan M.I.R., Anjum N.A., Masood A., Hussain S.J., Khan N.A. (2018). Jasmonates in plants under abiotic stresses: Crosstalk with other phytohormones matters. Environ. Exp. Bot..

[260] Wasternack C., Parthier B. (1997). Jasmonate-signalled plant gene expression. Trends Plant Sci..

[261] Anjum S.A., Wang L., Farooq M., Khan I., Xue L. (2011). Methyl Jasmonate-Induced Alteration in Lipid Peroxidation, Antioxidative Defence System and Yield in Soybean Under Drought. J. Agron. Crop Sci..

[262] Shan C., Zhou Y., Liu M. (2015). Nitric oxide participates in the regulation of the ascorbate-glutathione cycle by exogenous jasmonic acid in the leaves of wheat seedlings under drought stress. Protoplasma.

[263] Lehmann J., Atzorn R., Brückner C., Reinbothe S., Leopold J., Wasternack C., Parthier B. (1995). Accumulation of jasmonate, abscisic acid, specific transcripts and proteins in osmotically stressed barley leaf segments. Planta.

[264] Ghassemi-Golezani K., Farhangi-Abriz S. (2018). Foliar sprays of salicylic acid and jasmonic acid stimulate H^+^-ATPase activity of tonoplast, nutrient uptake and salt tolerance of soybean. Ecotoxicol. Environ. Saf..

[265] Ahmad P., Kumar A., Ashraf M., Akram N.A. (2012). Salt-induced changes in photosynthetic activity and oxidative defense system of three cultivars of mustard (*Brassica juncea* L.). Afr. J. Biotechnol..

[266] Abdelgawad Z., Khalafaallah A.A., Abdallah M. (2014). Impact of methyl jasmonate on antioxidant activity and some biochemical aspects of maize plant grown under water stress condition. Agric. Sci..

[267] Bandurska H., Stroiński A., Kubiś J. (2003). The effect of jasmonic acid on the accumulation of ABA, proline and spermidine and its influence on membrane injury under water deficit in two barley genotypes. Acta Physiol. Plant..

[268] Farooq M.A., Gill R.A., Islam F., Ali B., Liu H., Xu J., He S., Zhou W. (2016). Methyl jasmonate regulates antioxidant defense and suppresses arsenic uptake in *Brassica napus* L.. Front. Plant Sci..

[269] Poonam S., Kaur H., Geetika S. (2013). Effect of jasmonic acid on photosynthetic pigments and stress markers in *Cajanus cajan* (L.) Millsp. seedlings under copper stress. Am. J. Plant Sci..

[270] Sirhindi G., Mir M.A., Abd-Allah E.F., Ahmad P., Gucel S. (2016). Jasmonic acid modulates the physio-biochemical attributes, antioxidant enzyme activity, and gene expression in *Glycine max* under nickel toxicity. Front. Plant Sci..

[271] Ahmad P., Alyemeni M.N., Wijaya L., Alam P., Ahanger M.A., Alamri S.A. (2017). Jasmonic acid alleviates negative impacts of cadmium stress by modifying osmolytes and antioxidants in faba bean (*Vicia faba* L.). Arch. Agron. Soil Sci..

[272] Ali E., Hussain N., Shamsi I.H., Jabeen Z., Siddiqui M.H., Jiang L.-X. (2018). Role of jasmonic acid in improving tolerance of rapeseed (*Brassica napus* L.) to Cd toxicity. J. Zhejiang Univ. Sci. B.

[273] Yoon J.Y., Hamayun M., Lee S.-K., Lee I.-J. (2009). Methyl jasmonate alleviated salinity stress in soybean. J. Crop Sci. Biotechnol..

[274] Fedina I., Nedeva D., Georgieva K., Velitchkova M. (2009). Methyl Jasmonate Counteract UV-B Stress in Barley Seedlings. J. Agron. Crop Sci..

[275] Demkura P.V., Abdala G., Baldwin I.T., Ballaré C.L. (2010). Jasmonate-Dependent and -Independent Pathways Mediate Specific Effects of Solar Ultraviolet B Radiation on Leaf Phenolics and Antiherbivore Defense. Plant Physiol..

[276] Ahmad P., Hashem A., Abd-Allah E.F., Alqarawi A.A., John R., Egamberdieva D., Gucel S. (2015). Role of *Trichoderma harzianum* in mitigating NaCl stress in Indian mustard (*Brassica juncea* L.) through antioxidative defense system. Front. Plant Sci..

[277] Ahmad P., Sarwat M., Bhat N.A., Wani M.R., Kazi A.G., Tran L.S. (2015). Alleviation of cadmium toxicity in *Brassica juncea* L. (Czern. & Coss.) by calcium application involves various physiological and biochemical strategies. PLoS ONE.

[278] Carpena R.O., Vázquez S., Esteban E., Fernández-Pascual M., de Felipe M.R., Zornoza P. (2003). Cadmium-stress in white lupin: Effects on nodule structure and functioning. Plant Physiol. Biochem..

[279] Sakamoto A., Murata N. (2002). The role of glycine betaine in the protection of plants from stress: Clues from transgenic plants. Plant Cell Environ..

[280] Ahmad P., Abass Ahanger M., Nasser Alyemeni M., Wijaya L., Alam P., Ashraf M. (2018). Mitigation of sodium chloride toxicity in *Solanum lycopersicum* L. by supplementation of jasmonic acid and nitric oxide. J. Plant Interact..

[281] Nahar K., Hasanuzzaman M., Alam M.M., Rahman A., Suzuki T., Fujita M. (2016). Polyamine and nitric oxide crosstalk: Antagonistic effects on cadmium toxicity in mung bean plants through upregulating the metal detoxification, antioxidant defense and methylglyoxal detoxification systems. Ecotoxicol. Environ. Saf..

[282] Benavides M.P., Groppa M.D., Recalde L., Verstraeten S.V. (2018). Effects of polyamines on cadmium- and copper-mediated alterations in wheat (*Triticum aestivum* L.) and sunflower (*Helianthus annuus* L.) seedling membrane fluidity. Arch. Biochem. Biophy..

[283] Groppa M.a.D., Benavides M.a.P., Tomaro M.a.L. (2003). Polyamine metabolism in sunflower and wheat leaf discs under cadmium or copper stress. Plant Sci..

[284] Gális I., Šimek P., Narisawa T., Sasaki M., Horiguchi T., Fukuda H., Matsuoka K. (2006). A novel R2R3 MYB transcription factor NtMYBJS1 is a methyl jasmonate-dependent regulator of phenylpropanoid-conjugate biosynthesis in tobacco. Plant J..

[285] Shi J., Xie D., Qi D., Peng Q., Chen Z., Schreiner M., Lin Z., Baldermann S. (2019). Methyl Jasmonate-Induced Changes of Flavor Profiles During the Processing of Green, Oolong, and Black Tea. Front. Plant Sci..

[286] Ahmadi F.I., Karimi K., Struik P.C. (2018). Effect of exogenous application of methyl jasmonate on physiological and biochemical characteristics of *Brassica napus* L. cv. Talaye under salinity stress. S. Afr. J. Bot..

[287] Fischer C., Höll W. (1992). Food reserves of scots pine (*Pinus sylvestris* L.). Trees.

[288] El-Khallal S. (2001). Some physiological roles of jasmonic acid in adaptation of pea seedlings to salt stress. Egypt. J. Biotechnol..

[289] Ghoulam C., Foursy A., Fares K. (2002). Effects of salt stress on growth, inorganic ions and proline accumulation in relation to osmotic adjustment in five sugar beet cultivars. Environ. Exp. Bot..

[290] Harpreet K., Poonam S., Geetika S. (2013). Sugar accumulation and its regulation by jasmonic acid in *Brassica napus* L. under salt stress. J. Stress Physiol. Biochem..

[291] Garcıa-Mata C., Lamattina L. (2002). Nitric oxide and abscisic acid cross talk in guard cells. Plant Physiol..

[292] Cutler S.R., Rodriguez P.L., Finkelstein R.R., Abrams S.R. (2010). Abscisic acid: Emergence of a core signaling network. Ann. Rev. Plant Biol..

[293] Hartung W. (2010). The evolution of abscisic acid (ABA) and ABA function in lower plants, fungi and lichen. Funct. Plant Biol..

[294] Xu D., Yuan H., Tong Y., Zhao L., Qiu L., Guo W., Shen C., Liu H., Yan D., Zheng B. (2017). Comparative Proteomic Analysis of the Graft Unions in Hickory (*Carya cathayensis*) Provides Insights into Response Mechanisms to Grafting Process. Front. Plant Sci..

[295] Kumar R.M.S., Ji G., Guo H., Zhao L., Zheng B. (2018). Over-expression of a grafting-responsive gene from hickory increases abiotic stress tolerance in *Arabidopsis*. Plant Cell Rep..

[296] Ben Hassine A., Ghanem M.E., Bouzid S., Lutts S. (2009). Abscisic acid has contrasting effects on salt excretion and polyamine concentrations of an inland and a coastal population of the Mediterranean xero-halophyte species *Atriplex halimus*. Ann. Bot..

[297] Pattanagul W. (2011). Exogenous Abscisic Acid Enhances Sugar Accumulation in Rice. Asian J. Plant Sci..

[298] Sripinyowanich S., Klomsakul P., Boonburapong B., Bangyeekhun T., Asami T., Gu H., Buaboocha T., Chadchawan S. (2013). Exogenous ABA induces salt tolerance in indica rice (*Oryza sativa* L.): The role of OsP5CS1 and OsP5CR gene expression during salt stress. Environ. Exp. Bot..

[299] Karimi R., Ershadi A. (2015). Role of exogenous abscisic acid in adapting of ‘Sultana’grapevine to low-temperature stress. Acta Physiol. Plant..

[300] Ding W., Song L., Wang X., Bi Y. (2010). Effect of abscisic acid on heat stress tolerance in the calli from two ecotypes of *Phragmites communis*. Biol. Plant..

[301] Zhang L., Gao M., Hu J., Zhang X., Wang K., Ashraf M. (2012). Modulation Role of abscisic acid (ABA) on growth, water relations and glycinebetaine metabolism in two maize (*Zea mays* L.) cultivars under drought stress. Int. J. Mol. Sci..

[302] Pal M., Tajti J., Szalai G., Peeva V., Vegh B., Janda T. (2018). Interaction of polyamines, abscisic acid and proline under osmotic stress in the leaves of wheat plants. Sci. Rep..

[303] Sarafraz-Ardakani M.-R., Khavari-Nejad R.-A., Moradi F., Najafi F. (2014). Abscisic acid and cytokinin-induced osmotic and antioxidant regulation in two drought-tolerant and drought-sensitive cultivars of wheat during grain filling under water deficit in field conditions. Not. Sci. Biol..

[304] Kumar S., Kaushal N., Nayyar H., Gaur P. (2012). Abscisic acid induces heat tolerance in chickpea (*Cicer arietinum* L.) seedlings by facilitated accumulation of osmoprotectants. Acta Physiol. Plant..

[305] Lee T.M., Lur H.S., Chu C. (1997). Role of abscisic acid in chilling tolerance of rice (*Oryza sativa* L.) seedlings.: II. Modulation of free polyamine levels. Plant Sci..

[306] Marcinska I., Czyczylo-Mysza I., Skrzypek E., Grzesiak M.T., Janowiak F., Filek M., Dziurka M., Dziurka K., Waligorski P., Juzon K. (2013). Alleviation of osmotic stress effects by exogenous application of salicylic or abscisic acid on wheat seedlings. Int. J. Mol. Sci..

[307] Shevyakova N., Musatenko L., Stetsenko L., Vedenicheva N., Voitenko L., Sytnik K., Kuznetsov V.V. (2013). Effects of abscisic acid on the contents of polyamines and proline in common bean plants under salt stress. Russ. J. Plant Physiol..

[308] Planchet E., Verdu I., Delahaie J., Cukier C., Girard C., Morere-Le Paven M.C., Limami A.M. (2014). Abscisic acid-induced nitric oxide and proline accumulation in independent pathways under water-deficit stress during seedling establishment in *Medicago truncatula*. J. Exp. Bot..

[309] Verslues P.E., Bray E.A. (2006). Role of abscisic acid (ABA) and Arabidopsis thaliana ABA-insensitive loci in low water potential-induced ABA and proline accumulation. J. Exp. Bot..

[310] Burbulis N., Jonytienė V., Kuprienė R., Blinstrubienė A., Liakas V. (2010). Effect of abscisic acid on cold tolerance in *Brassica napus* shoots cultured in vitro. J. Food Agric. Environ..

[311] Yang C., Zhou Y., Fan J., Fu Y., Shen L., Yao Y., Li R., Fu S., Duan R., Hu X. (2015). *SpBADH* of the halophyte *Sesuvium portulacastrum* strongly confers drought tolerance through ROS scavenging in transgenic *Arabidopsis*. Plant Physiol. Biochem..

[312] Golestan Hashemi F.S., Ismail M.R., Rafii M.Y., Aslani F., Miah G., Muharam F.M. (2018). Critical multifunctional role of the betaine aldehyde dehydrogenase gene in plants. Biotechnol. Biotechnol. Equip..

[313] Toumi I., Moschou P.N., Paschalidis K.A., Bouamama B., Salem-Fnayou A.B., Ghorbel A.W., Mliki A., Roubelakis-Angelakis K.A. (2010). Abscisic acid signals reorientation of polyamine metabolism to orchestrate stress responses via the polyamine exodus pathway in grapevine. J. Plant Physiol..

[314] Hassanein R.A., Hassanein A.A., El-din A.B., Salama M., Hashem H.A. (2009). Role of jasmonic acid and abscisic acid treatments in alleviating the adverse effects of drought stress and regulating trypsin inhibitor production in soybean plant. Aust. J. Basic Appl. Sci..

[315] Jimenez-Bremont J.F., Ruiz O.A., Rodriguez-Kessler M. (2007). Modulation of spermidine and spermine levels in maize seedlings subjected to long-term salt stress. Plant Physiol. Biochem..

[316] Rakitin V.Y., Prudnikova O., Rakitina T.Y., Karyagin V., Vlasov P., Novikova G., Moshkov I. (2009). Interaction between ethylene and ABA in the regulation of polyamine level in *Arabidopsis thaliana* during UV-B stress. Russ. J. Plant Physiol..

[317] Alcázar R., Cuevas J.C., Patron M., Altabella T., Tiburcio A.F. (2006). Abscisic acid modulates polyamine metabolism under water stress in *Arabidopsis thaliana*. Physiol. Plant..

[318] Liu J., Jiang M.Y., Zhou Y.F., Liu Y.L. (2005). Production of polyamines is enhanced by endogenous abscisic acid in maize seedlings subjected to salt stress. J. Integr. Plant Biol..

[319] Hanzawa Y., Imai A., Michael A.J., Komeda Y., Takahashi T. (2002). Characterization of the spermidine synthase-related gene family in *Arabidopsis thaliana*. FEBS Lett..

[320] Urano K., Yoshiba Y., Nanjo T., Igarashi Y., Seki M., Sekiguchi F., Yamaguchi-Shinozaki K., Shinozaki K. (2003). Characterization of Arabidopsis genes involved in biosynthesis of polyamines in abiotic stress responses and developmental stages. Plant Cell Environ..

[321] Gurmani A., Bano A., Khan S., Din J., Zhang J. (2011). Alleviation of salt stress by seed treatment with abscisic acid (ABA), 6-benzylaminopurine (BA) and chlormequat chloride (CCC) optimizes ion and organic matter accumulation and increases yield of rice (*Oryza sativa* L.). Aust. J. Crop Sci..

[322] Gusta L., Trischuk R., Weiser C. (2005). Plant cold acclimation: The role of abscisic acid. J. Plant Growth Regul..

[323] Pelleschi S., Guy S., Kim J.Y., Pointe C., Mahe A., Barthes L., Leonardi A., Prioul J.L. (1999). Ivr2, a candidate gene for a QTL of vacuolar invertase activity in maize leaves. Gene-specific expression under water stress. Plant Mol. Biol..

[324] Trouverie J., Chateau-Joubert S., Thevenot C., Jacquemot M.P., Prioul J.L. (2004). Regulation of vacuolar invertase by abscisic acid or glucose in leaves and roots from maize plantlets. Planta.

[325] Trouverie J., Thévenot C., Rocher J.P., Sotta B., Prioul J.L. (2003). The role of abscisic acid in the response of a specific vacuolar invertase to water stress in the adult maize leaf. J. Exp. Bot..

[326] Farooq U., Bano A. (2006). Effect of abscisic acid and chlorocholine chloride on nodulation and biochemical content of *Vigna radiata* L. under water stress. Pak. J. Bot..

[327] Kumar S., Kaur G., Nayyar H. (2008). Exogenous application of abscisic acid improves cold tolerance in chickpea (*Cicer arietinum* L.). J. Agron. Crop Sci..

[328] Cheng Z., Jin R., Cao M., Liu X., Chan Z. (2016). Exogenous application of ABA mimic 1 (AM1) improves cold stress tolerance in bermudagrass (*Cynodon dactylon*). Plant Cell Tissue Organ Cult..

[329] An Y., Zhou P., Liang J. (2014). Effects of exogenous application of abscisic acid on membrane stability, osmotic adjustment, photosynthesis and hormonal status of two lucerne (*Medicago sativa* L.) genotypes under high temperature stress and drought stress. Crop Pasture Sci..

[330] Dias M.C., Oliveira H., Costa A., Santos C. (2014). Improving elms performance under drought stress: The pretreatment with abscisic acid. Environ. Exp. Bot..

